# Health beliefs and parental responses to human papillomavirus vaccination in the Middle East: a theory-informed narrative synthesis guided by the health belief model

**DOI:** 10.3389/fpubh.2026.1853938

**Published:** 2026-10-23

**Authors:** Ahmad Abuzoor, Md. Shafiqur Rahman Jabin, Cor Jonker

**Affiliations:** 1 Faculty of Health Studies, University of Bradford, Bradford, United Kingdom; 2 Department of Medicine and Optometry, Linnaeus University, Kalmar, Sweden

**Keywords:** health belief model, human papillomavirus, Middle Eastern, parents

## Abstract

**Background:**

Human papillomavirus (HPV) is the most common sexually transmitted infection in women and the primary cause of cervical cancer (CC). HPV vaccination and screening programs have significantly reduced CC incidence and mortality worldwide, preventing up to 70% of HPV-related CC and 90% of genital warts. Developing effective vaccination strategies is therefore essential.

**Objectives:**

This study systematically synthesizes the attitudes and health beliefs of Middle Eastern (ME) parents regarding HPV vaccination, examines parental vaccine uptake, and assesses uptake among their children.

**Methods:**

This systematic review followed PRISMA guidelines. PubMed/MEDLINE, Web of Science, CINAHL, and Google Scholar were searched for studies published between January 2006 and May 2025. Thirty-three studies were included. A theory-informed narrative synthesis of predominantly quantitative evidence was conducted, with deductive mapping of findings to the health belief model (HBM) constructs. The review protocol was registered in PROSPERO (CRD42025645551).

**Results:**

The review included 32 quantitative cross-sectional studies and one qualitative study, involving 18,170 participants. Findings were organized according to HBM constructs, including perceived susceptibility, severity, barriers, benefits, self-efficacy, and cues to action. Parents generally demonstrated limited knowledge of HPV and vaccination. Major barriers included safety concerns, cost, limited access, lack of healthcare provider recommendations, and insufficient information. Cultural and systemic factors were also associated with vaccine uptake.

**Conclusion:**

HPV-related knowledge in ME countries remains insufficient and is frequently associated with vaccination attitudes and acceptance across the included studies. Improving uptake requires targeted educational interventions, healthcare provider engagement, public health campaigns, financial support, and policy-level initiatives. Strengthening awareness and addressing structural barriers are critical for enhancing vaccination acceptance. Self-efficacy, where directly reported, appeared to be associated with more favorable vaccine attitudes; however, direct evidence for parental self-efficacy as a distinct HBM construct was limited across the included studies and should not be overstated.

**Systematic review registration:**

https://www.crd.york.ac.uk/PROSPERO/myprospero/CRD42025645551.

## Introduction

The prevalence of sexually transmitted diseases (STDs) is on the rise in Middle Eastern (ME) countries, which had previously been reported to have low rates compared to the rest of the world (1). This rise is attributed to rapid life changes due to globalization and shifts in sexual behavior among the younger generation in the ME (2). Human papillomavirus (HPV) is the most common STD in women worldwide and the leading cause of cervical cancer (CC) and anogenital cancer in both men and women. In some ME countries, such as Oman and the United Arab Emirates (UAE), CC is the second most common cancer among women (3). About 80% of sexually active individuals are infected with the human papillomavirus (HPV). There are more than 200 different strains of HPV, with around 40 transmitted through sexual activities. Most HPV infections are asymptomatic, with 80% resolving within 1 year. Nearly 70% of HPV-related cancers are caused by the two most common strains, HPV 16 and 18, which pose significant cancer risks (4). HPV vaccines are now available and can potentially reduce the incidence of CC and other anogenital cancers. HPV is a major cause of CC. The best prevention against HPV infection, and therefore CC, is the HPV vaccine. Early vaccination, before the first sexual activity, remains the gold standard in primary prevention of CC and can be a powerful means of protecting oneself from the virus and disease (4). Presently, the approved HPV vaccine formulations include three bivalent vaccines (offering protection against two strains of HPV), two quadrivalent vaccines (providing protection against four strains), which together account for about 90 percent of CC cases, and one nonavalent vaccine (protecting against nine strains) (4). Only two ME countries, including the UAE and Saudi Arabia, have effectively introduced the HPV vaccine through national programs (5). However, most studies indicate that people primarily favor vaccines (6). Some studies show a positive attitude toward vaccination due to fear of HPV, CC, and genital warts, but there are also concerns about encouraging sexual promiscuity among children. Only 20 countries included boys in their HPV immunization programs in 2017 (7). Parents play a crucial role in preventing HPV in their children through their decision-making process. However, low knowledge and understanding of HPV and the vaccine among parents and vaccinated girls have been reported, and decisions are often made without sufficient awareness of relevant issues.

A recent systematic review found that immigrant parents in Australia generally have low knowledge, attitudes, and perceptions about HPV-related diseases and vaccines and negative attitudes toward HPV vaccines (8). However, to our knowledge, no systematic review has specifically synthesized findings from studies conducted within ME countries among parents residing in the region. The review by Netfa et al. (8), the closest related work, examined HPV-related knowledge and attitudes among immigrant Arabic-speaking parents living in Australia, a fundamentally different cultural and health-system context. The present review therefore addresses a distinct gap by focusing exclusively on parents living within ME countries, where health policy, access infrastructure, and sociocultural norms differ substantially from those in diaspora settings. Despite a preliminary search of the Cochrane Database of Systematic Reviews, International Prospective Registry of Systematic Reviews (PROSPERO), Joanna Briggs Institute (JBI) Evidence Synthesis, and Campbell Systematic Reviews, no current systematic reviews or scoping reviews were found.

This study aimed to systematically synthesize parents’ attitudes and health beliefs regarding HPV vaccination in ME countries. Its objectives are to explore factors associated with parental HPV vaccine acceptance and uptake, assess the level of actual vaccination uptake reported by parents in ME countries, and examine how government policies in ME countries influence parental vaccine decision-making.

The review questions were as follows:

- What factors of controversy exist for parents (attitude, knowledge, perceptions, and health beliefs) regarding HPV vaccines in ME countries?
- How can the health belief model (HBM) be used to interpret parental acceptance and uptake of HPV vaccination in ME countries?
- How are government policies in ME countries involved in advocating for the acceptance of HPV vaccines?

## Methods

The systematic review followed the Preferred Reporting Items for Systematic Reviews and Meta-Analyses (PRISMA) guidelines (9). This systematic review uses a theory-informed narrative synthesis to examine how parents in Middle Eastern countries view HPV vaccination, including their attitudes, knowledge, perceptions, and health beliefs. Most of the findings come from quantitative cross-sectional surveys and one qualitative study, and the results are interpreted using the HBM. Quantitative survey findings, such as frequencies and associations related to knowledge, attitudes, perceived barriers, and intentions, were extracted in textual form and subsequently transformed into qualitative codes and themes. The resulting themes were deductively mapped onto constructs of the HBM, including perceived susceptibility, severity, benefits, barriers, cues to action, self-efficacy, and modifying variables. This method clarifies the analytic process, which flows from survey data to narrative results, then codes and themes, and ultimately to HBM constructs. The protocol for this systematic review was registered with the International Prospective Registry of Systematic Reviews (PROSPERO) under the registration number CRD42025645551.

### Search strategy and critical appraisal

Studies selected for retrieval were assessed by two independent reviewers (AA as the primary reviewer) for methodological validity before inclusion, using the JBI Critical Appraisal Checklist for Analytical Cross-Sectional Studies for the 32 quantitative studies (10) and the Critical Appraisal Skills Programme (CASP) qualitative checklist for the single qualitative study (11). This procedure revealed minor discrepancies, which were resolved by consensus through discussions among the research group (CJ served as an arbitrator). Four electronic databases, namely, PubMed/MEDLINE, Web of Science, CINAHL, and Google Scholar, were systematically searched to identify studies between January 1, 2006, and May 2025, because the first licensed HPV vaccine was introduced in 2006 (12). The complete search strategy and keywords are provided in Table 1.

**Table 1:** Search term strategy and keywords.

Database	Date searched	Search string	Filters applied
PubMed/MEDLINE	May 2025	(“papillomavirus vaccines”[MeSH] OR “HPV vaccine*” OR “human papillomavirus vaccin*” OR “HPV vaccin*”) AND (“parents”[MeSH] OR “parent*” OR “mother*” OR “father*” OR “guardian*”) AND (“attitude*” OR “perception*” OR “knowledge” OR “acceptan*” OR “uptake” OR “willingness” OR “health belief*”) AND (“Middle East*” OR “Bahrain” OR “Cyprus” OR “Egypt” OR “Iran” OR “Iraq” OR “Israel” OR “Jordan” OR “Kuwait” OR “Lebanon” OR “Oman” OR “Palestine” OR “Qatar” OR “Saudi Arabia” OR “Syria” OR “Turkey” OR “United Arab Emirates” OR “Yemen”)	English language; 2006–2025
Web of Science	May 2025	(TS = (“HPV vaccin*” OR “human papillomavirus vaccin*” OR “papillomavirus vaccine*”)) AND (TS = (“parent*” OR “mother*” OR “father*” OR “guardian*”)) AND (TS = (“attitude*” OR “perception*” OR “knowledge” OR “acceptan*” OR “uptake” OR “willingness”)) AND (TS = (“Middle East*” OR “Bahrain” OR “Egypt” OR “Iran” OR “Iraq” OR “Israel” OR “Jordan” OR “Kuwait” OR “Lebanon” OR “Oman” OR “Qatar” OR “Saudi Arabia” OR “Syria” OR “Turkey” OR “UAE” OR “Yemen”))	English language; 2006–2025
CINAHL	May 2025	(MH “Papillomavirus Vaccines” OR TI “HPV vaccin*” OR AB “human papillomavirus vaccin*”) AND (MH “Parents” OR TI “parent*” OR AB “mother*” OR AB “father*”) AND (TI “attitude*” OR AB “perception*” OR AB “acceptan*” OR AB “uptake” OR AB “willingness”) AND (TI “Middle East*” OR AB “Saudi Arabia” OR AB “Turkey” OR AB “Lebanon” OR AB “Jordan” OR AB “Iran” OR AB “Bahrain” OR AB “UAE” OR AB “Oman” OR AB “Egypt”)	English language; 2006–2025
Google Scholar	May 2025	“HPV vaccine” OR “human papillomavirus vaccine” AND “parents” AND “Middle East” OR “Saudi Arabia” OR “Turkey” OR “Lebanon” OR “Jordan” OR “Iran”	First 20 pages of results screened (200 records); English language; 2006–2025

### Inclusion criteria

The inclusion criteria adopted for this systematic review were based on the Population, Phenomenon of Interest, and Context (PICo) framework. The review considered studies whose populations included parents or primary guardians of children under 18 years of age in ME countries. Although the initial eligibility criteria allowed for the inclusion of studies recruiting HPV carriers or patients with cervical cancer that reported parental outcomes separately, no eligible studies specifically reporting this subgroup with distinct parental data were identified; therefore, all included studies and synthesized findings concern parents or guardians of children under 18. The phenomena of interest were parents’ knowledge, perceptions, and health beliefs regarding their children’s HPV vaccination. Furthermore, the context of this review involved studies conducted in the ME countries. The ME countries, according to the world atlas, include 17 countries: Bahrain, Cyprus, Egypt, Iran, Iraq, Israel, Jordan, Kuwait, Lebanon, Oman, Palestine, Qatar, Saudi Arabia, the Syrian Arab Republic, Turkey, the United Arab Emirates, and Yemen (13). The systematic review included peer-reviewed studies published in English. Quantitative observational studies and qualitative studies were included in the current review.

For clarification, the included studies had to recruit participants who were parents or primary guardians of children under 18, and the main outcomes had to concern their knowledge, attitudes, beliefs, willingness, or behaviors regarding HPV vaccination for their children rather than for themselves. Additionally, studies of general adult samples (e.g., university students, unmarried adults, health professionals) were excluded unless parents of minors were reported as a distinct subgroup with separate data in Table 2.

**Table 2:** Inclusion and exclusion criteria.

Criteria category	Inclusion criteria	Exclusion criteria
Article type	Peer-reviewed primary research articles, quantitative and qualitative studies. Full-text articles available	Non-peer-reviewed articles, commentaries, conference abstracts without peer review, or grey literature (e.g., reports, theses). Articles without full text access or incomplete data.
Language	Studies published in English.	Studies published in languages other than English.
Population	Parents or primary guardians of children under 18 years of age residing in ME countries. No eligible studies specifically reporting HPV carrier or cervical cancer patient data as a distinct parental subgroup were identified	Studies that focus on general adult groups, such as university students, unmarried adults, or healthcare workers, are excluded unless they provide separate data and analysis for parents of minors.
Context	Studies conducted in ME countries	Studies conducted outside the specific ME countries.
Publication period	Studies published between 1 January 2006 and May 2025.	Studies published before January 2006 and after May 2025.

### Data extraction and synthesis of results

Data from the final list of included studies were extracted using a data extraction form developed by the authors, using a standardized approach in a tabular format. This form consisted of the following fields: author name, types of studies, publication year, journal, country; study aims and objectives, methods and study design, sample size, and variables related to parents’ uptake of HPV vaccination.

Findings were synthesized using a theory-informed narrative synthesis with inductive coding and deductive mapping to HBM constructs. In the first stage, key results on parents’ knowledge, perceptions, attitudes, willingness, and reported vaccination uptake were extracted from each study in textual form and inductively coded into recurring descriptive patterns (e.g., safety concerns, cost barriers, and physician recommendations). Similar codes were grouped into broader descriptive themes. In the second stage, these themes were deductively mapped onto the predefined HBM constructs of perceived susceptibility, perceived severity, perceived benefits, perceived barriers, cues to action, and self-efficacy. This process clearly distinguishes the analytical approach from a formal qualitative thematic synthesis and is consistent with the predominantly quantitative evidence base. The explicit content of included studies was considered at the semantic level, without assumptions about latent underpinnings. Because most included studies did not explicitly operationalize HBM constructs in their design, this mapping represents an interpretive, retrospective process undertaken by the review authors and should not be regarded as equivalent to direct measurement of HBM constructs. Quantitative cross-sectional survey results and qualitative findings were first summarized narratively for each study and then synthesized across studies into higher-order themes.

For quantitative studies, key results on parents’ knowledge, perceptions, attitudes, willingness, and reported vaccination uptake were extracted from the results sections and tables in textual form. These findings were then transformed into qualitative codes (e.g., low HPV knowledge, safety concerns, cost barriers, physician recommendations, vaccine uptake) based on the direction and strength of reported frequencies or associations, as well as the authors’ interpretations. Similar codes across studies were grouped into broader descriptive themes such as vaccine safety and side-effect concerns, financial and access barriers, and the influence of healthcare providers.

In the second stage, descriptive themes were deductively mapped onto HBM constructs, including perceived susceptibility, perceived severity, perceived benefits, perceived barriers, cues to action, self-efficacy, and modifying variables. When a theme corresponded to more than one construct, the dominant construct was assigned following team discussion. Safety concerns were classified as perceived barriers in all cases, as this is their most direct HBM classification; reclassification as low self-efficacy was applied only where the original study explicitly assessed parental confidence in completing the vaccination process. Other themes that did not fit any HBM construct (e.g., concerns about HPV vaccination, modifying variables in HPV uptake, and the attitude of parents toward HPV vaccination uptake) were retained under the category of other factors.

The primary reviewer first coded the retrieved data, followed by the secondary reviewer, who independently coded a purposive subset of studies from different countries and outcome categories. The coding framework and construct mapping were iteratively improved through discussions until agreement was reached. If necessary, a third reviewer was consulted to resolve any disagreements. After reaching an agreement, the final coding methodology was applied to all the studies.

### Quality appraisal

None of the studies were excluded from this systematic review based on the quality appraisal results. The 32 quantitative cross-sectional studies were assessed using the JBI Critical Appraisal Checklist for Analytical Cross-Sectional Studies, which is considered the most appropriate tool for this study design. The single qualitative study (14) was assessed using the CASP qualitative checklist (11). The JBI checklist evaluates the clarity of aims, sampling strategy, description of setting and participants, data analysis, identification of confounders, and reliability of outcome measurement. The tool did not provide a way to objectively score and categorize the studies but assessed the quality of the included studies in three broad areas: validity of the study results, outcomes, and whether the study results are helpful locally. All studies reported clearly focused aims and objectives. Similarly, all studies described participant recruitment, and there was minimal risk of reporting bias in all studies except one. A qualitative study, however, did not describe reflexivity in its methodology, so it was unclear if there was any bias in reporting (14). Similarly, all studies used acceptable outcome measures; however, due to their qualitative nature, they did not report any specific outcome measures (14). Whether confounding factors were taken into consideration was unclear in six studies (15–20). Sample size justifications were not provided in 10 studies (19, 21–29). The precision and accuracy of findings were evident in all included studies. Similarly, all studies reported implications. Generalizability was questionable in 10 studies (14, 16, 17, 21, 22, 24–26, 29, 30) (Table 3).

**Table 3:** Quality appraisal of included studies—JBI Checklist for Analytical Cross-Sectional Studies (studies 1–32) and CASP Qualitative Checklist (study 33).

NO	Author and year of publication	Clearly focused aim	Recruitment of participants acceptable	Minimal chances of reporting bias	Outcome measures acceptable	Confounding factors taken into consideration	Sample size justified	Precision of findings	Accuracy of findings	Generalizability	Implications
1	Alherz et al. (2024) (21)	Yes	Yes	Yes	Yes	Yes	Unclear	Yes	Yes	Unclear	Yes
2	Elissa et al. (2024) (31)	Yes	Yes	Yes	Yes	Yes	Yes	Yes	Yes	Yes	Yes
3	Zakhour et al. (2023) (16)	Yes	Yes	Yes	Yes	Unclear	Yes	Yes	Yes	Unclear	Yes
4	Abuduxike et al. (2022) (22)	Yes	Yes	Yes	Yes	Yes	Unclear	Yes	Yes	Unclear	Yes
5	Akca et al. (2022) (32)	Yes	Yes	Yes	Yes	Yes	Yes	Yes	Yes	Yes	Yes
6	Çelik et al. (2021) (33)	Yes	Yes	Yes	Yes	Yes	Yes	Yes	Yes	Yes	Yes
7	Alnaeem et al. (2023) (23)	Yes	Yes	Yes	Yes	Yes	Unclear	Yes	Yes	Yes	Yes
8	Bin Alamir et al. (2024) (34)	Yes	Yes	Yes	Yes	Yes	Yes	Yes	Yes	Yes	Yes
9	Seven et al. (2015) (17)	Yes	Yes	Yes	Yes	Unclear	Yes	Yes	Yes	Unclear	Yes
10	Alaamri et al. (2023) (35)	Yes	Yes	Yes	Yes	Yes	Yes	Yes	Yes	Yes	Yes
11	Alkalash et al. (2022) (30)	Yes	Yes	Yes	Yes	Yes	Yes	Yes	Yes	Unclear	Yes
12	Alshehri et al. (2023) (15)	Yes	Yes	Yes	Yes	Unclear	Yes	Yes	Yes	Yes	Yes
13	Alhusayn et al. (2022) (36)	Yes	Yes	Yes	Yes	Yes	Yes	Yes	Yes	Yes	Yes
14	Ghojazadeh et al. (2012) (24)	Yes	Yes	Yes	Yes	Yes	Unclear	Yes	Yes	Unclear	Yes
15	Tobaiqy et al. (2023) (37)	Yes	Yes	Yes	Yes	Yes	Yes	Yes	Yes	Yes	Yes
16	Azh et al. (2021) (38)	Yes	Yes	Yes	Yes	Yes	Yes	Yes	Yes	Yes	Yes
17	Alnafisah et al. (2019) (18)	Yes	Yes	Yes	Yes	Unclear	Yes	Yes	Yes	Yes	Yes
18	Alzahrani et al. (2017) (12)	Yes	Yes	Yes	Yes	Yes	Yes	Yes	Yes	Yes	Yes
19	Anfinan (2019) (25)	Yes	Yes	Yes	Yes	Yes	Unclear	Yes	Yes	Unclear	Yes
20	Husain et al. (2019) (39)	Yes	Yes	Yes	Yes	Yes	Yes	Yes	Yes	Yes	Yes
21	Jassim et al. (2018) (40)	Yes	Yes	Yes	Yes	Yes	Yes	Yes	Yes	Yes	Yes
22	Alrajeh and Alshammari (2020) (26)	Yes	Yes	Yes	Yes	Yes	Unclear	Yes	Yes	Unclear	Yes
23	Ortashi et al. (2014) (27)	Yes	Yes	Yes	Yes	Yes	Unclear	Yes	Yes	Yes	Yes
24	Saqer et al. (2017) (19)	Yes	Yes	Yes	Yes	Unclear	Unclear	Yes	Yes	Yes	Yes
25	Kilic et al. (2012) (28)	Yes	Yes	Yes	Yes	Yes	Unclear	Yes	Yes	Yes	Yes
26	Shibli and Rishpon (2019) (29)	Yes	Yes	Yes	Yes	Yes	Unclear	Yes	Yes	Unclear	Yes
27	Levent et al. (2021) (41)	Yes	Yes	Yes	Yes	Yes	Yes	Yes	Yes	Yes	Yes
28	Alsaad et al. (2012) (42)	Yes	Yes	Yes	Yes	Yes	Yes	Yes	Yes	Yes	Yes
29	Abou El-Ola et al. (2018) (20)	Yes	Yes	Yes	Yes	Unclear	Yes	Yes	Yes	Yes	Yes
30	Hendaus et al. (2021) (43)	Yes	Yes	Yes	Yes	Yes	Yes	Yes	Yes	Yes	Yes
31	Al Alawi et al. (2023) (44)	Yes	Yes	Yes	Yes	Yes	Yes	Yes	Yes	Yes	Yes
32	Alyahya et al. (2024) (45)	Yes	Yes	Yes	Yes	Yes	Yes	Yes	Yes	Yes	Yes
33	Jradi and Bawazir (2019) (14)	Yes	Yes	Unclear	Unclear	Yes	Yes	Yes	Yes	Unclear	Yes

## Results

### Study selection

Figure 1 illustrates the study selection procedure using a PRISMA flowchart. After a thorough search, we identified 381 reports through electronic database searches and 6 reports from records obtained through snowballing. The final search of the selected databases was conducted in May 2025, and 330 studies were obtained after removing duplicates. We excluded 249 studies based on the screening of article titles, abstracts, and keywords. The remaining 81 studies underwent full-text screening for eligibility. This process resulted in 48 articles being excluded after full-text review for the following reasons: no explicitly reported parental HPV-related outcome (knowledge, attitude, belief, willingness, or uptake; *n* = 31); population not meeting inclusion criteria, such as studies recruiting healthcare workers, students, or general adults without a distinct parental subgroup (*n* = 10); study conducted outside the specified ME countries (*n* = 4); and study design not meeting inclusion criteria, such as narrative reviews or conference abstracts (*n* = 3). Thirty-three articles were included in the final synthesis.

**Figure 1:**
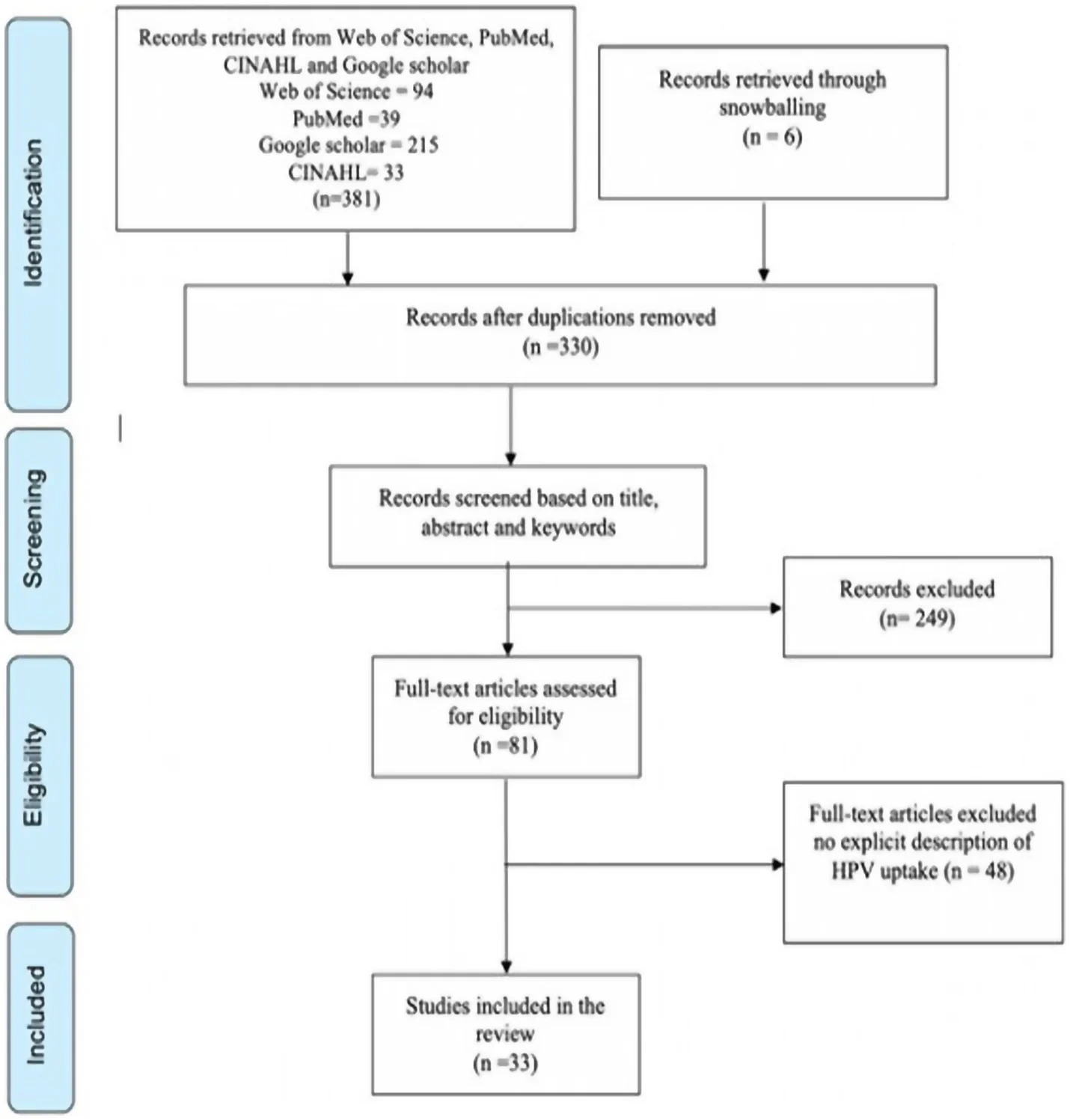
PRISMA flow chart of the articles/records screening process HPV vaccine uptake.

### Included study characteristics

Of the 33 included studies, 32 were quantitative observational (cross-sectional) studies, while only one was a qualitative study (14). The included studies were published between 2012 and 2024. The majority of the studies were published in Saudi Arabia (*n* = 13) (12, 14, 15, 18, 21, 23, 25, 30, 34–37) followed by Turkey (*n* = 5) (17, 28, 32, 33, 41) and Lebanon (*n* = 3) (16, 20, 31). All included studies assessed parents’ knowledge, attitudes, and perceptions regarding HPV and its vaccination. The sample size varied across the studies. Of the 32 cross-sectional studies, 5 used online questionnaires for data collection (23, 30, 31, 35, 37), while the remaining 27 studies used self-administered questionnaires for data collection. The qualitative research included focus group discussions and semi-structured individual interviews for data collection (14). A total of 18,170 participants were included in this systematic review. Most of the participants were parents (mostly mothers), according to the cross-sectional surveys (Table 4).

**Table 4:** Summary of the studies included.

NO	Author and publication year	Countries	Study design	Aim of the study	Participants and data collection	Key findings	Suggestions and recommendations
1	Alherz et al. (2024) (21)	Saudi Arabia	Cross-sectional study	To evaluate HPV vaccine acceptability among parents and daughters.	A total of 390 parents were invited. Participants self-administered a questionnaire to collect data.	– HPV vaccination was expected to be given to daughters by 60% of the parents. – Parents participating lacked knowledge. – Parental education levels were substantially correlated with parental knowledge of HPV infection and its vaccination; parents with a bachelor’s degree or more (54.9%) showed better awareness of HPV than parents with just a high school or middle school education (41.9%) and (41.5%), respectively.	– The approval rate for the HPV vaccination will be positively impacted by the Saudi Ministry of Health’s initiatives to increase awareness among the population. – To increase the degree of acceptability of the HPV vaccination, parents’ knowledge must be raised.
2	Elissa et al. (2024) (31)	Lebanon	Cross-sectional study	To assess parents’ intent and awareness level regarding HPV vaccination in Lebanon	Recruitment included 392 parents. Participants completed an electronic questionnaire to collect data.	– The intent to be vaccinated against HPV showed a favorable correlation with demographic factors. It also correlated with insurance coverage and knowledge of HPV and its vaccine.	– To increase the vaccination rate among teenagers, government representatives should also make sure that the HPV vaccination is available at no charge – To increase parental knowledge, comprehension, and attitudes around HPV, the research emphasized the need for educational campaigns. This will help parents accept vaccinations for their children.
3	Zakhour et al. (2023) (16)	Lebanon	Cross-sectional study	To explain how information, beliefs, and actions about vaccines generally affect actions associated with the HPV vaccine.	A total of 306 parents participated Data were collected by a survey.	– There was no apparent influence from cost, cultural, or religious views. – Around 60% of parents were not giving the vaccine or intending to provide the HPV vaccination to their children. – Parents’ main reasons for reluctance to vaccinate were the absence of suggestions from pediatricians and insufficient information on the vaccine. – 7.5% of parents opposed to the HPV vaccination said it was unnecessary, while 13% believed it to be unsafe.	– Doctors should also have the training and authority they need to offer the HPV vaccination more firmly and regularly. – Parental education on the significance of the vaccine and its proven safety and effectiveness should be prioritized, regardless of gender.
4	Abuduxike et al. (2022) (22)	Cyprus	Cross-sectional study	To examine parental awareness and knowledge of HPV infection, associated illnesses, and HPV vaccinations	227 parents participated Data were obtained using an interviewer-administered questionnaire.	– The following factors significantly predicted better knowledge of HPV vaccination: having a higher level of education, being a woman, being aged 35 and above, and being employed.	– Increasing vaccination acceptance requires a greater emphasis on successful educational programs targeted at sociodemographic populations.
5	Akca et al. (2022) (32)	Turkey	Cross-sectional study	The purpose was to evaluate Turkish mothers’ attitudes and knowledge about HPV infection and vaccination.	330 parents participated. The questionnaire was used.	– Working in healthcare, having a higher education, and economic status were all strongly linked to awareness of what causes HPV infections and severity. – The HPV vaccination was unknown to 71.5% of mothers.	– Medical professionals may use the findings of this research to identify parents who may be vaccine-hesitant and start tailored education to encourage timely children’s immunization.
6	Çelik et al. (2021) (33)	Turkey	– Cross-sectional study	To evaluate parents’ knowledge, attitudes, and views regarding HPV and its vaccination.	1,000 parents were surveyed. Data used from the questionnaire	– HPV vaccinations were given to children by just two of their parents. – Only 22% knew the importance of HPV vaccination, and 26.9% knew about HPV. Media outlets were parents’ main source of information. – 5.5% of children’s parents said their pediatrician and the general doctor told them.	– Improved communication between parents and the medical community can raise the idea that immunization should be given at a young age, and the family’s knowledge about HPV and its vaccine – The transmission methods of HPV should be addressed and communicated by specialists as part of the sexual education course in schools.
7	Alnaeem et al. (2023) (23)	Saudi Arabia	Cross-sectional study	To assess parents’ knowledge and acceptance of HPV vaccination.	The authors surveyed 380 parents via an online questionnaire.	– The primary obstacles to immunization were worries about adverse effects and vaccine efficacy. The sociodemographic variables had a substantial impact on acceptability and knowledge – 67.1% of parents said their children had not had the vaccination, and 62.9% said their physicians had not brought it up. – The HPV vaccination was only known to 46.6% of the subjects. – Acceptance of the vaccination was influenced by MOH approval and trust in its efficacy.	– Structured awareness efforts and increased interaction between healthcare professionals and parents are key to improving vaccination uptake.
8	Bin Alamir et al. (2024) (34)	Saudi Arabia	Cross-sectional study	To evaluate the knowledge and awareness in depth of parents about HPV infections, their related illness concerns, and the advantages of the vaccine	281 parents participated A self-administered questionnaire	– Due to differences in vaccination refusal rates and communication barriers regarding the HPV vaccine, marital status influenced vaccine-related choices. – HPV vaccine knowledge and self-efficacy were more significant among females. – Those with more education, such as those with a university degree, supported mandatory vaccinations more.	– To address the complex issues regarding the HPV vaccine and raise population vaccine knowledge generally, the study results recommended a variety of multimodal approaches, such as policy implications, concentrated public awareness campaigns, as well as learning programs – To bridge knowledge gaps and increase HPV vaccination rates, study findings emphasized the need for medical professionals and specially designed training programs.
9	Seven et al. (2015) (17)	Turkey	Cross-sectional study	To assess parental readiness to permit vaccination against HPV for their children aged 10–13 years.	Data were collected from 368 parents through a survey (questionnaire).	– Only 14.4% of mothers and 15.5% of fathers expressed an interest in receiving vaccinations for their daughters, compared to 21.6% of mothers and 22.4% of fathers who expressed an interest in getting immunized for their boys. – Of mothers and fathers, 24.5 and 21.2%, respectively, said they knew about the vaccination, while just 26.9 and 25.0%, respectively, reported knowing about HPV.	– Initiatives to raise awareness and knowledge of HCPs regarding HPV and its vaccination should be supported, and both sexes should be educated.
10	Alaamri et al. (2023) (35)	Saudi Arabia	Cross-sectional study	Assessing the obstacles and enablers of the HPV vaccine and related aspects among daughters’ parents.	Researchers recruited 947 parents via a web-based questionnaire to collect data.	– Compared to parents who are unaware of the link between HPV infection and cervical carcinoma, those who are aware of it were 1.794 times more likely to agree to the vaccination. – Mothers were 1.627 times more knowledgeable about HPV and its vaccination than fathers, primarily when they were working with a higher salary, had greater levels of education, and were under 40 years of age.	Clinicians must be engaged, as their advice significantly affects parents’ decisions to immunize their children. – Several strategies, such as curriculum modifications, educational activities, and social media campaigns, should be implemented.
11	Alkalash et al. (2022) (30)	Saudi Arabia	Cross-sectional study	To evaluate the attitudes and knowledge on the HPV vaccination	Participated: 343 parents Data were collected using an online questionnaire	– The HPV vaccination was known to parents in roughly 32.9%, and doctors were the most prevalent provider of this information. 75.2% of respondents hesitated to be vaccinated because they believed they were not at risk.	– Women should be educated about the risk of cervical cancer and the value of screening programs, according to the research. – Saudi Arabs’ awareness of cervical cancer and the advantages of screening and vaccination would rise with a well-thought-out health education program. It is recommended that primary healthcare practitioners educate their patients and customers about HPV vaccination and urge them to vaccinate their daughters.
12	Alshehri et al. (2023) (15)	Saudi Arabia	Cross-sectional study	To evaluate attitudes and knowledge about HPV vaccination.	773 parents participated Surveys were used to collect data.	– 38.5% of parents had a good understanding of HPV, 15.4% had some knowledge, and 46.1% had no knowledge. −11.9% did not plan to get vaccinated, 43.6% were unsure, and 44.5% intended to be vaccinated. Knowledge level was significantly correlated with participants’ nationality, education, age, work sector, monthly income, and whether they were fathers or mothers.	– Culturally sensitive health promotion programs for the HPV vaccination should highlight its advantages and potential risks for women. – Effective education efforts addressing parents and children in schools may increase understanding and beliefs around HPV. – Even though HPV vaccinations are safe and effective, additional comprehensive approaches could be required to raise HPV vaccination rates throughout the country.
13	Alhusayn et al. (2022) (36)	Saudi Arabia	Cross-sectional study	To assess parents’ knowledge and attitudes regarding the HPV vaccine.	296 parents were examined using a questionnaire	– A history of HPV vaccination was substantially correlated with employment position, while gender and educational attainment were significant determinants of HPV vaccination uptake. – Parents are uninformed about the HPV vaccine in 70.6% of cases. – For unvaccinated responders who had no plans to get the HPV vaccination, the reasons for reluctance were of statistically noteworthy influence.	Strong awareness campaigns are required to improve HPV-related education and boost Saudi parental acceptability of HPV vaccinations.
14	Ghojazadeh et al. (2012) (24)	Iran	Cross-sectional study	The knowledge and attitudes of parents regarding HPV should be examined	358 parents participated used questionnaire.	– Mothers had better average knowledge of HPV infection compared to fathers, with their level of education and age having an essential influence on parental knowledge. – 76% of parents were unaware of the seriousness of HPV infection. – Of the parents with knowledge, 36% had learned about it online, while others had done so by researching medical resources.	– Given society’s educational state, it will be more beneficial to use public media to expand the database of knowledge of HPV, since most parents learn about the virus from medical information and via the Internet. – The research found a significant level of unawareness about HPV infection, emphasizing the need to educate all adults in the community.
15	Tobaiqy et al. (2023) (37)	Saudi Arabia	Cross-sectional study	Investigating parents’ attitudes, beliefs, and knowledge of the HPV vaccination	500 parents were recruited Data were collected using an online questionnaire	– About parents, just 11% were aware of HPV. – The primary rationale for declining the immunization was insufficient awareness about the need for HPV vaccination. –The participants proposed many strategies to boost vaccine acceptability, including government systems, instructional lectures in educational institutions, and social media knowledge. – 94% of parents were against vaccinating their children against HPV, and (96.8%) were unaware of the vaccine.	– To decrease the chances of cervical cancer, programs to increase knowledge, acceptability, and get to HPV vaccinations for specific populations must be developed. Additional national research using health education theoretical frameworks, such as the HBM, will improve our understanding of parents’ vaccine hesitation, perceived advantages, and vaccination challenges.
16	Azh et al. (2021) (38)	Iran	Cross-sectional study	To determine what influences a mother’s decision to vaccinate her teenage children against HPV	126 mothers were recruited Self-administered questionnaires were used to gather data.	– Parents’ understanding, level of education, and income levels were all linked to their plans to get their children vaccinated against HPV. – The exorbitant expense of vaccinations, economic challenges, and insufficient awareness were significant obstacles to HPV immunization.	– Health professionals should make parents and teenagers aware of the advantages of the HPV vaccine. This procedure ought to be endorsed by governments.
17	Alnafisah et al. (2019) (18)	Saudi Arabia	Cross-sectional study	To evaluate female attitudes and knowledge on vaccines and cervical carcinoma screening.	2,220 mothers were recruited Data were obtained by questionnaire.	– When their girls reached school age, 70.8% of mothers consented to get immunized. – Just 3.2% of respondents thought HPV was a risk factor. – According to 68.6% of women, cervical cancer might be avoided with the HPV vaccination.	– Awareness programs are essential to enhance knowledge and increase attendance, therefore dispelling unfavorable attitudes and beliefs around the HPV vaccine.
18	Alzahrani et al. (2017) (12)	Saudi Arabia	Cross-sectional study	To investigate the parental perspective and knowledge of the HPV vaccine.	278 parents were recruited Data were gathered through a self-administered survey.	– 94.69% supported HPV vaccination of their daughters before marriage. – A number of respondents said that insufficient support from their spouses obstructed the vaccination of their daughters. – Of both genders’ individuals, 78.30% did not know that HPV vaccination can prevent cervical cancer.	– It is necessary to educate children and parents about the importance of the HPV vaccine. Educating people about HPV and its vaccination is the responsibility of the MOH and HCPs.
19	Anfinan (2019) (25)	Saudi Arabia	Cross-sectional study	To evaluate parental attitudes and views on HPV vaccination rates	The number of parents contacted was 2000. A self-administered questionnaire was used to gather data.	– (77.6%) of parents consented to vaccinating their children against HPV. – Concerns were around the safety and expenses associated with the HPV vaccine.	– Targeted educational efforts are necessary to stimulate doctors’ active involvement in decreasing cervical cancer and HPV. – To create a successful preventative approach, such initiatives should clarify the HPV vaccine by revealing its safety, effectiveness, and availability, as well as provide helpful information about the immunization process.
20	Husain et al. (2019) (39)	Bahrain	Cross-sectional study	To assess the degree of knowledge and perceptions of HPV for parents	A questionnaire was utilized to obtain data from the 408 parents contacted.	– 76% of individuals expressed a willingness to get the vaccination. – Of the participants, 38.5% said that children and their parents should decide together whether to vaccinate against HPV, and over 90% accepted that teenagers should be given information about the virus. – A primary concern regarding the HPV vaccine was the side effects, efficacy, and cost.	– Education on HPV and the function of vaccination must be given to the Bahraini society. – The analysis identified substantial deficiencies in HPV knowledge, which might be addressed in forthcoming informational initiatives. – Insufficient knowledge among males may be a specific difficulty and requires prioritization.
21	Jassim et al. (2018) (40)	Bahrain	Cross-sectional study	To evaluate mothers’ attitudes, practices, and knowledge about the HPV vaccination	Data were acquired from 300 mothers using a completed questionnaire.	– (90.9%) of participants expressed their willingness to permit children to receive HPV vaccinations. – Barriers to the HPV vaccine include cultural sensitivities and religious beliefs. – A few individuals also mentioned how they would feel embarrassed and worried if male medical professionals performed their examinations.	– Establishing a long-term public relations campaign on preventing cervical cancer is necessary, and it also highlights the need for a based-on populations cervical screening program, based-on populations cervical screening program implemented all over the country in primary healthcare facilities.
22	Alrajeh and Alshammari (2020) (26)	Saudi Arabia	Cross-sectional study	To evaluate the knowledge of mothers’ perspectives and awareness of HPV vaccination	326 mothers were recruited The questionnaire was used to collect data.	– Only 29.1% of women thought that they would have their daughters vaccinated for HPV. – 21% of individuals know about the HPV vaccination. – The primary obstacle is insufficient knowledge and awareness of HPV and its vaccine.	– In light of the recent reforms in the Saudi health system that prioritize prevention, health policymakers need to establish a nationwide preventative plan to boost the adoption of the HPV vaccination and cervical screening tests. – Using behavioral and informational interventions, a variety of channels, and relevant multimedia may raise awareness and comprehension of the vaccination.
23	Ortashi et al. (2014) (27)	United Arab Emirates	Cross-sectional study	To assess the variables that affect mothers’ acceptance of the HPV vaccine	640 mothers were recruited The questionnaire was used to collect data.	– Only 9.8% of mother reported that they had vaccinated their daughters for HPV. – (87.4%) women said that recommending HPV vaccination to their family or friends is important. – One of the participants cited obstacles to the HPV vaccine as being sensitive to culture. – A few individuals believed that the HPV vaccination was unsafe.	– The knowledge and attitude of the parents can be improved with mass media campaigns and healthcare-derived interventions, which need to be formulated in the UAE.
24	Saqer et al. (2017) (19)	United Arab Emirates	Cross-sectional study	To evaluate parental knowledge and attitudes toward HPV vaccinations among daughters.	400 parents were recruited The questionnaire was used to collect the data.	– 76.6 percent of parents said they would be willing to vaccinate their daughters against HPV. – Those surveyed reported worries over the safety and harmful effects of HPV vaccination and believed that their acceptance would increase if the MOH recommended the HPV vaccination.	– Before suggesting that the vaccine be included in the UAE immunization schedule, a thorough socioeconomic assessment of its effectiveness is required.
25	Kilic et al. (2012) (28)	Turkey	Cross-sectional study	Assess the uniformity of parental perspectives on HPV vaccination among teenage females.	A survey was used to collect data from the 301 recruited parents.	– 45.5% of the mothers and 44.9% of the fathers expressed support for the HPV vaccination of their daughters. – Lack of vaccination information was the most frequent excuse given for refusal to be vaccinated.	– Adolescent girls, the target group, as well as their parents, are thought to need enough and accurate information on the vaccine.
26	Shibli and Rishpon (2019) (29)	Israel	Cross-sectional study	To evaluate mothers’ knowledge and attitudes about vaccinations and identify variables influencing their consent to the HPV vaccine.	Researchers enrolled 313 parents and gathered information through a self-administered survey.	– Of mothers who agreed to their child’s uptake of the vaccination, only 65.8% – Fewer than 50% of the mothers perceived the immunization as safe. – Of the three factors that continued to be substantially linked with the mother’s agreement to the HPV vaccination, the knowledge degree was adversely related to the mother’s assent to the vaccination. – The attitude level and self-efficacy were found to be positively related to the mother’s acceptance of the vaccine	A special program should address beliefs and promote the vaccine’s advantages and safety to improve attitudes toward HPV vaccination and raise acceptance rates. These programs’ focus must be tailored to the industry in which they are executed, namely, raising Arab parents’ understanding and awareness of vaccination while focusing on raising Jewish parents’ attitudes toward it.
27	Levent et al. (2021) (41)	Turkey	Cross-sectional study	To examine parents’ knowledge of HPV vaccination for adolescents and their attitudes toward vaccination.	229 parents participated via questionnaire	– 57.0% of parents said that the expense of vaccinations was one of the factors contributing to their unfavorable attitudes about vaccinations. – Additional causes include insufficient awareness about vaccines and a lack of familial cancer history. – 20% discuss vaccination protection, while 3.4% discuss vaccine adverse effects.	– To improve the vaccination rate, all parents and adolescents applying to any medical facility should be made aware of vaccinations. Educating the public about raising immunization rates is insufficient. – The vaccines should be added to the calendar by the government, and support should be supplied to ensure that clinicians are continually informed about this subject.
28	Alsaad et al. (2012) (42)	Syria	Cross-sectional study	To test the acceptance of mothers of girls of HPV vaccination.	A questionnaire was employed to acquire data from 345 mothers.	– 236 parents (68.4%) agreed to vaccinate their daughters against HPV. – 31.6% denied immunization, while 44.0% requested further information before making a choice. There were 15.6% who were worried about vaccine adverse effects and 13.8 who believed that their daughter was not at risk for cervical cancer.	– Before any HPV vaccine program is implemented, more must be done to educate Syrians. Public health campaigns should prioritize educating mothers, the general public, and healthcare professionals.
29	Abou El-Ola et al. (2018) (20)	Lebanon	Cross-sectional study	To assess mothers’ understanding of HPV vaccination	1,193 mothers were recruited The questionnaire was used.	The main reason is that 14.4% blamed the high cost of vaccines for the lack of vaccination. – 22.9% believed it was an ineffective vaccine, and 11.7% did not get vaccinated because they worried about its safety. Only 0.9% expected their daughters to have sex before marriage. Only 0.9% expected their daughters to have sex before marriage. – There are many obstacles to vaccination, but the primary one is mothers’ misunderstanding about HPV, the vaccine, and preventative strategies.	– To increase HPV vaccination rates, awareness campaigns and a combined strategy that addresses the obstacles found would be advised.
30	Hendaus et al. (2021) (43)	Qatar	Cross-sectional study	To determine parental attitudes about HPV vaccination	The study included 334 parents who completed the survey.	– 77% of participants felt confident administering a vaccination to the children that would protect them against genital cancer. – A significant percentage of parents have a favorable view of the HPV vaccination.	– Educating and raising awareness of parents’ attitudes and beliefs is crucial for effective community health intervention.
31	Al Alawi et al. (2023) (44)	Oman	Cross-sectional study	To assess parents’ attitudes, understanding, and acceptance of the HPV vaccine	1,403 parents were recruited by questionnaire	– 84.6% needed confirmation that the vaccination would protect against HPV infection, whereas only 37.4% thought it was safe. – There was a significant difference in parents’ beliefs about the optimal age for HPV vaccination. – Dedicated support was demonstrated for HPV education in schools, which includes preventative techniques as part of programs that educate students about reproductive and sexual health.	– The research emphasized the need for Oman to start a nationwide HPV vaccination program and conduct HPV education at the community and school levels.
32	Alyahya et al. (2024) (45)	Jordan	Cross-sectional study	To investigate parents’ understanding and perceptions about HPV and HPV vaccinations	386 parents were recruited Data were collected by questionnaire	– 39.9% of parents thought that the HPV vaccination was helpful. – 35.5% of participants said they trusted the HPV vaccine’s effectiveness. – Findings show that female individuals had higher belief ratings than male participants about HPV vaccines.	– Community pharmacies may facilitate the expanded coverage and awareness of vaccines, and it is imperative to concentrate on the parents of adolescents in order to facilitate vaccination efforts.
33	Jradi and Bawazir (2019) (14)	Saudi Arabia	Qualitative study (focus group discussions and individual interviews)	To determine how mothers feel about being vaccinated against HPV and examine their knowledge of the virus	A total of 77 mothers participated in focus groups. By Semi-structured interviews	– Parents consented to provide their children with the HPV vaccination contingent upon good enough evidence of its safety. – Some of the participants showed concerns about male guardianship and their inability to make decisions independently regarding the vaccination of their daughters. – Lack of availability and knowledge is one of the barriers to getting the HPV vaccine. In addition, the sensitive nature of the subject matter posed obstacles to their practice, as it was subject to cultural restrictions.	– The focus must be on educating and raising women’s knowledge of the risk factors for cervical cancer and the need for screening programs and vaccination.

### Study findings

The results were structured around the core HBM constructs of perceived susceptibility, perceived severity, perceived benefits, perceived barriers, cues to action, and self-efficacy, with sociodemographic characteristics treated as modifying variables. Because most included studies were not designed to measure HBM constructs directly, this mapping is interpretive and retrospective; it should not be regarded as equivalent to direct measurement of HBM constructs. Additionally, other factors emerged from the included studies and are reported under ‘Other factors’. Throughout this review, a critical distinction is maintained between two outcome types: (1) willingness or intention to vaccinate, defined as a parent’s self-reported readiness or agreement to have their child receive the HPV vaccine; and (2) actual vaccination uptake, defined as parent-reported receipt of at least one dose of the HPV vaccine by their child. These outcomes are not interchangeable; willingness reflects attitudinal readiness, while uptake reflects documented behavior. Where studies reported both or either outcome, this is clearly noted in the text and in Table 5.

**Table 5:** Outcomes of willingness to vaccinate and actual vaccination uptake.

Study (Author, Year)	Country	Outcome type	Outcome definition	Data source
Ortashi et al. (2014) (27)	United Arab Emirates	Actual uptake	Parent reports daughter has received HPV vaccine (any dose)	Self-reported questionnaire
Çelik et al. (2021) (33)	Turkey	Actual uptake	HPV vaccination received by child (very low—2 of 1,000 parents sampled)	Self-reported questionnaire
Shibli and Rishpon (2019) (29)	Israel	Actual uptake and willingness	Mothers’ agreement for HPV vaccination and reported receipt of vaccine by child	Self-reported questionnaire
Alherz et al. (2024) (21)	Saudi Arabia	Willingness	Parent’s willingness to vaccinate daughter against HPV	Self-reported questionnaire
Alnafisah et al. (2019) (18)	Saudi Arabia	Willingness	Mothers’ consent to vaccinate daughters when they reach school age	Self-reported questionnaire
Anfinan (2019) (25)	Saudi Arabia	Willingness	Parent consented to vaccinate child against HPV	Self-reported questionnaire
Jassim et al. (2018) (40)	Bahrain	Willingness	Mothers’ willingness to permit HPV vaccination for their child	Self-reported questionnaire
Husain et al. (2019) (39)	Bahrain	Willingness	Parents’ willingness to vaccinate their child against HPV	Self-reported questionnaire
Elissa et al. (2024) (31)	Lebanon	Willingness	Parent willingness to vaccinate child against HPV	Self-reported questionnaire
Zakhour et al. (2023) (16)	Lebanon	Willingness	Parents’ intention not to vaccinate their child against HPV	Self-reported questionnaire
Alyahya et al. (2024) (45)	Jordan	Willingness	Parents’ trust in and intention toward HPV vaccine for their child	Self-reported questionnaire
Kilic et al. (2012) (28)	Turkey	Willingness	Mothers’ and fathers’ support for their daughter’s HPV vaccination	Self-reported questionnaire

W = willingness/intention to vaccinate; U = actual reported uptake. Where studies reported both outcomes, both are noted. Willingness consistently exceeded actual uptake where both were measured, indicating a willingness–uptake gap across the region.

### Perceived susceptibility to HPV and vaccination measure

Perceived susceptibility, as a core HBM construct, refers to a parent’s subjective belief about the likelihood of their child contracting an HPV infection (46). It is important to distinguish between genuine perceived susceptibility, where a parent expresses a belief about personal or child-specific risk, and knowledge or awareness of HPV susceptibility, which reflects factual understanding rather than subjective risk perception. Similarly, associations between sociodemographic characteristics (education, income, age, nationality) and knowledge of susceptibility represent modifying variable effects and are not themselves HBM perceptions. The findings below are therefore organized into: (a) genuine perceived susceptibility beliefs, and (b) knowledge and awareness related to susceptibility. Thirteen studies examined this construct.

#### HPV as a risk factor for other health conditions

This was a widely discussed theme in most of the included studies. Gender was significantly associated with HPV infection rates, increasing susceptibility to other comorbidities like CC (44). Parents often worry that their children are not immune to HPV risks and may indeed be infected throughout their life course (29), but studies show low perceived risks. Parents who perceive HPV as a risk factor prioritize vaccination for their children (18). In contrast, those who do not perceive HPV as a risk factor are less concerned about it (26). Low-risk susceptibility participants assumed their children were not engaged in premarital sex (20). Parents who trust their spouses kept them free from STDs (39). CC’s untreatable nature was also associated with perceived susceptibility to HPV vaccination, as respondents said there was no reason to vaccinate or conduct screening tests because they believed that it is incurable (14).

#### Vaccination education and knowledge

Several included studies reported findings that reflect knowledge or awareness of HPV susceptibility rather than a parent’s subjective belief about personal or child-specific risk. Research indicated that most parents lacked knowledge about HPV and its transmission, meaning they could not adequately assess the risk to their children (21, 33). Studies reporting that higher educational attainment was associated with greater awareness of HPV susceptibility (34) represent modifying variable effects on knowledge rather than direct evidence of perceived susceptibility as an HBM construct. Similarly, findings that susceptibility awareness correlated with income level, nationality, age, and employment status (15, 18) reflect sociodemographic associations with knowledge and should be interpreted as modifying variable associations rather than as evidence of the HBM perceived susceptibility construct itself. Inadequate knowledge of HPV risk was reported as a primary reason for non-vaccination among 21.1% of respondents (Table 6).

**Table 6:** Themes related to perceived susceptibility.

Study	HBM concept: Perceived susceptibility	Themes
Alnafisah et al.’s (2019) (18)	Few participating parents believe HPV is a risk factor for their children. Parents (82.9%) who perceived HPV as a prevalent risk factor were significantly more in favor of vaccinating their children compared to those (68.9%) who did not perceive HPV as a harmful infection.	My children are not at risk.
Alrajeh et al. (2020) (26)	People think they are not susceptible to the virus, so they do not worry about Pap smear tests or HPV vaccines.	My children are not at risk
Abou El-Ola et al. (2018) (20)	Mothers genuinely believe that their daughters or future sons-in-law will never be involved in premarital sex and are thus not worried about HPV infection.	My children are not at risk because they will not get involved in premarital sex
Al Alawi et al. (2023) (44)	Parents mentioned that men and women can get HPV at rates of 78.5 and 82.9%, respectively. Furthermore, 58.5% of men and 78.3% of women believed that HPV caused cancer and abnormal cervical smears in women	Men and women may get HPV HPV can cause cervical cancer in women.
Alyahya et al. (2024) (45)	Marriage at a young age and smoking are associated with higher HPV infection and cervical cancer.	Early marriage and smoking increase the risk of HPV infection.
Alherz et al. (2024) (21)	Despite the general lack of participants’ knowledge of HPV and its vaccination, educated participants had basic knowledge of susceptibility.	Knowledge of susceptibility—awareness of HPV risk associated with educational attainment (modifying variable effect on knowledge, not direct perceived susceptibility).
Çelik et al. (2021) (33)	26.9% of parents have information about HPV susceptibility, and most do not have sufficient knowledge that their children might be susceptible to it.	Limited knowledge and awareness of HPV susceptibility knowledge finding, not subjective risk belief.
Bin Alamir et al. (2024) (34)	Higher-educated parents, such as those with a university degree, had greater knowledge regarding susceptibility. A survey indicated that 46.1% of parents had inadequate awareness of HPV susceptibility, 15.4% had a moderate level of understanding, and 38.5% demonstrated a proficient level of knowledge.	Higher knowledge of susceptibility was observed among more educated parents (sociodemographic modifying variable—see Modifying Variables section)
Alshehri et al. (2023) (15)	Knowledge of susceptibility was highly correlated with respondents’ nationality, educational stage, salary, and whether they were the father or mother.	Knowledge of susceptibility correlated with nationality, education, income, and parental role—modifying variable associations with knowledge, not direct perceived susceptibility.
Alnafisah et al.’s (2019) (18)	Inadequate knowledge about HPV (21.1% of the research participants), as well as its perceived ineffectiveness, was the main reason behind not getting vaccinated against HPV. 58.5% of the people also thought that they were unlikely to be exposed to the virus, so they did not believe in getting vaccinated against it.	Inadequate knowledge of HPV risk was the primary reason for non-vaccination among 21.1% of participants—a knowledge barrier, not perceived susceptibility.
Husain et al. (2019) (39)	People in their research distrust a newly introduced vaccine, and they also believe that their spouses will not give them STDs, which is why they refuse to get the vaccine.	My children are not at risk
Jradi and Bawazir (2019) (14)	There was no point in getting the vaccine or the screening test.	There was no sense in receiving the vaccination or cervical cancer screening test at all because cervical cancer is untreatable.
Shibli and Rishpon (2019) (29)	47.6% of the parents in the research believed that their children are susceptible to contracting HPV at any time in their lives.	My children are at risk of HPV infection.

Entries labeled as ‘knowledge/awareness findings’ reflect factual understanding of HPV susceptibility rather than parents’ subjective beliefs about personal or child-specific risk. Sociodemographic associations are modifying variable effects on knowledge. Where the original study did not explicitly measure perceived susceptibility as an HBM construct, findings are mapped interpretively.

### Perceived severity of HPV and vaccination measures

Perceived severity refers to a parent’s subjective belief about how serious the consequences of HPV infection would be for their child, including the likelihood of developing cervical cancer or other HPV-related complications (46). It is important to distinguish this from knowledge or awareness of HPV severity, which reflects factual understanding rather than subjective belief about personal consequences. Most included studies measured parents’ knowledge of HPV severity rather than directly assessing their subjective beliefs about seriousness as an HBM construct. Associations between sociodemographic variables (income, education, occupation, nationality) and knowledge of severity are modifying variable effects and should not be equated with perceived severity as an HBM perception. Thirteen studies examined this construct, and findings are organized accordingly.

#### Awareness of HPV severity and its consequences

Across the 13 studies examined under this construct, the dominant finding was limited awareness of HPV-related consequences among ME parents. It is important to note at the outset that most of these studies measured knowledge or awareness of HPV severity—that is, whether parents had factual information about the seriousness of HPV—rather than directly assessing their subjective belief about how serious the consequences would be for their own child. These are analytically distinct: the HBM predicts that it is the subjective appraisal of seriousness, not factual knowledge alone, that motivates health behavior. Findings reflecting knowledge of severity are labeled accordingly in Table 7 and should not be equated with the HBM construct of perceived severity.

**Table 7:** Evaluated perceived severity from studies.

Study	HBM concept: Perceived severity	Themes
Seven et al. (2015) (17)	Only 26.9% of mothers and 25.0% of fathers had some knowledge of HPV; thus, only a minor proportion of parents had sufficient knowledge of HPV. However, interestingly, those who had knowledge of HPV demonstrated greater concerns, which showed that they had knowledge of its severity.	Limited knowledge and awareness of HPV severity—knowledge finding.
Zakhour et al. (2023) (16)	Parents with knowledge of HPV were concerned about the severity of HPV infection.	Awareness of HPV severity associated with parental knowledge–knowledge finding.
Ghojazadeh et al. (2012) (24)	The severity of the infection caused by HPV was unknown to 76% of parents, as were the complications associated with it. Interestingly, parents with knowledge about HPV severity reported greater motivation for vaccinations for their children.	76% of parents were unaware of HPV severity—limited knowledge finding; knowledge associated with motivation to vaccinate.
Alaamri et al. (2023) (35)	Parents who understand the correlation are 1.794 times more likely to agree to the vaccine if they have a connection between HPV and cervical carcinoma.	Parental understanding of HPV–cancer link associated with 1.794 × higher likelihood of vaccine acceptance—closest to genuine perceived severity in this construct.
Hendaus et al. (2021) (43)	Of the participants, 77% felt at ease administering a vaccination to their kids that would protect them against malignancy. Some factors influence the knowledge of parents about the severity of HPV.	Knowledge of HPV severity was associated with willingness to vaccinate—knowledge finding.
Akca et al. (2022) (32)	Educational status, parental employment in healthcare, and income level were strongly associated with knowledge of HPV infection severity.	Perceived severity was associated with parental knowledge; education and employment are sociodemographic modifying variables
Ghojazadeh et al. (2012) (24)	76% of parents were unaware of the severity of HPV infection. Among the knowledgeable parents, 36% acquired their knowledge from online sources, while others obtained it from medical sources. Mothers’ knowledge of HPV was greater than that of fathers’, and both higher education and age significantly influenced parents’ understanding of HPV.	Limited knowledge of HPV severity—76% of parents were unaware; knowledge source and education are modifying variables.
Alshehri et al. (2023) (15)	A study indicated that severity knowledge was associated with the monthly income level, the parent’s gender (father or mother), degree of education, and work position.	Severity awareness was associated with knowledge; income, education, and employment were noted as sociodemographic correlates.
Elissa et al. (2024) (31)	A favorable association was observed between “The intent of getting vaccinated for HPV” and awareness of the severity of an infection with HPV. In the same way, knowledge regarding severity was contingent upon the children’s compliance with all necessary vaccinations, the father’s educational background, information on the HPV vaccination, parental vaccination against HPV, the family’s income, health insurance regarding the HPV vaccine, and the nationality of the mother.	Knowledge and awareness of HPV severity and the HPV-cancer link; sociodemographic factors noted as modifying variables.
Zakhour et al. (2023) (16)	The absence of recommendations from pediatricians and the perception that insufficient information was available on the vaccine were the predominant reasons for the lack of knowledge about the severity of infecting children with HPV.	Absence of pediatrician recommendations was associated with limited knowledge of HPV severity—knowledge barrier
Alnaeem et al. (2023) (23)	Most parents claimed that their physicians did not communicate the severity of the HPV infection to them. Knowledge of severity was strongly impacted by sociodemographic characteristics (education, gender, age).	Perceived severity was associated with parental knowledge; sociodemographic characteristics were modifying variables.
Bin Alamir et al. (2024) (34)	Mothers exhibited comparatively more knowledge about the severity of HPV infection than fathers. Higher-educated people had greater knowledge of the seriousness of the infection caused by HPV.	Mothers had greater knowledge of HPV severity than fathers—knowledge finding; education is a modifying variable.
Alkalash et al. (2022) (30)	The most common obstacle to vaccination was their conviction that they were not at risk of harm, demonstrating that parents had insufficient information regarding the seriousness of infection with HPV.	Parents’ low risk perception linked to insufficient knowledge of HPV severity—knowledge barrier.

Most entries in this table reflect knowledge or awareness of HPV severity rather than direct measurement of parents’ subjective beliefs about seriousness as an HBM construct. Where the original study did not explicitly measure perceived severity as an HBM perception, findings are mapped interpretively. Sociodemographic associations (education, income, occupation, nationality) represent modifying variable effects on knowledge.

The finding most closely approximating a genuine perceived severity effect came from Alaamri et al. (35), in which parents who understood the link between HPV and cervical cancer were 1.794 times more likely to accept the vaccine (24). Here, awareness of consequences translated into a measurable shift in vaccination acceptance, the mechanism the HBM predicts. In the remaining studies, findings reflected knowledge deficits rather than subjective severity appraisal: Ghojazadeh et al. (24) reported that approximately 76% of parents were unaware of the severity of HPV infection—a knowledge finding, not a direct measure of perceived seriousness (11).

Across countries, mothers consistently demonstrated greater awareness of HPV severity than fathers, and this difference was moderated by education, income, and employment status (24, 34); these findings represent modifying variable effects on knowledge rather than evidence of the perceived severity construct itself. The persistent absence of healthcare provider communication was identified across multiple studies as the primary structural reason for limited severity awareness (16, 23), pointing to a cue-to-action deficit at the system level rather than a deficit in perceived severity per se. These patterns collectively reinforce the importance of examining HBM constructs in combination rather than in isolation.

### Perceived barriers to HPV vaccination uptake

Researchers found that fear of barriers, such as religion, culture, lack of support, and insufficient information about vaccines and associated costs, is a significant reason for not changing health behaviors toward HPV, as some of the major themes explaining perceived barriers to HPV (Table 8).

**Table 8:** Major themes explaining perceived barriers to the HPV vaccine.

Study	HBM concept: Perceived barriers	Themes
Ortashi et al. (2014) (27), Saqer et al. (2017) (19), Jassim et al. (2018) (40)	One of the critical barriers to HPV vaccination of children was religion and cultural sensitivity.	Religion and culture are obstacles to HPV vaccine uptake
Alzahrani et al. (2017) (12), Alnafisah et al. (2019) (18)	A lack of the husband’s support or knowledge relating to Pap smear tests and HPV vaccinations	Lack of support
Jradi and Bawazir (2019) (14)	The lack of privacy or withholding of information about HPV vaccines was also an obstacle to receiving the HPV vaccine	Lack of HPV vaccine information
Husain et al. (2019) (39)	Parents were worried that education on the vaccination would “encourage sexual promiscuity among children”	Lack of HPV vaccine information
Alrajeh et al.’s (2020) (26)	The main reason for the negative responses about the HPV vaccine and getting Pap smear tests was an absence of knowledge	Lack of HPV vaccine information
Anfinan (2019) (25)	Absence of information on the vaccines, lack of understanding regarding their safety, and insecurity about their efficacy also act as barriers to people getting vaccinated	Lack of HPV vaccine information
Alsaad et al. (2012) (42)	A study reports that low awareness of the HPV vaccine is due to the fact that people were unaware that this virus causes cervical cancer or that cervical cancer can be avoided.	Lack of HPV vaccine information
Kilic et al. (2012) (28)	84.1% of mothers and 79.9% of fathers expressed a lack of understanding of the primary rationale for the refusal to immunize their girls against HPV.	Lack of HPV vaccine information
Husain et al. (2019) (39)	38.5% of parents believe that HPV vaccination is a decision made jointly by parents and children. Moreover, the cost of vaccination is a primary obstacle.	Cost barriers
Alzahrani et al. (2017) (12), Anfinan (2019) (25)	Studies reported that most of their research participants would like the vaccine to be free and stated that the price of vaccination is a constraint for people who cannot afford it	Cost barriers
Levent et al. (2021) (41)	57% of parents also expressed their unwillingness to vaccinate their child against HPV because of economic issues, mainly the cost.	Cost barriers
Hussain et al. (2019) (6)	The participating parents expressed concern about the cost of the vaccine.	Cost barriers
Ortashi et al. (2014) (27)	31.0% of women in their research expressed that they are usually not very concerned about their health, which is why they do not want to get the vaccine.	The vaccine was not considered important
Jradi and Bawazir (2019) (14), Ortashi et al. (2014) (27), Seven et al. (2015) (17), Azh et al. (2021) (38), Alhusayn et al. (2022) (36), Alaamri et al. (2023) (35)	Parents’ or research participants’ general lack of certainty about the safety of HPV vaccines, their lack of awareness, as well as the unavailability of HPV vaccines in healthcare facilities, lack of guidelines and recommendations for the vaccine, lack of time to get vaccinated, and poor patient-provider communication are also barriers to HPV vaccination uptake.	Lack of safety of the vaccine Vaccine unavailability in healthcare facilities Lack of recommendations Lack of time to get the vaccine
Alnafisah et al. (2019) (18)	People being unaware of where they can get Pap smear tests or an HPV vaccine, people not having enough support to do it, and having social difficulties were a few reasons they disagreed with getting the vaccine.	Lack of recommendations Vaccine is not important
Shibli and Rishpon (2019) (29)	A lack of appropriate information for the general public about the vaccine’s safety is why people are unwilling to consent.	Lack of HPV vaccine information
Alsaad et al. (2012) (42)	Only 18% knew about HPV, and 50% of them found out about it from television.	Lack of HPV vaccine information
Al Alawi et al. (2023) (44)	The study found that parents and HCPs lacked awareness regarding HPV infection and vaccinations. Approximately 62% of participants had no understanding of vaccine safety. 71.5% of respondents were concerned about the complications of the vaccine, while 84.6% wanted evidence that it would prevent HPV infection	Lack of HPV vaccine information Lack of safety information about the vaccine
Alyahya et al. (2024) (45)	66.7% of parents who are in favor of the HPV vaccine lacked awareness. Also, the financial issue was a barrier for 65.5% of parents, even though they are in favor of HPV; 65.5% or 57.4% are not.	Lack of HPV vaccine information No safety information about the vaccine
Zakhour et al. (2023) (16)	Lack of access or availability of the vaccine was a barrier for parents who were in favor of the HPV vaccine, accounting for 31.9%.	Lack of access to the vaccine
Alnaeem et al. (2023) (23)	Apprehensions over complications and vaccine efficacy were the primary obstacles to immunization. Factors affecting adoption included support from the MOH and confidence in the vaccine’s efficacy.	Lack of safety of the vaccine Lack of recommendations
Alkalash et al. (2022) (30)	The main obstacle to immunization was their belief that they did not belong at risk.	Lack of HPV vaccine information
Bin Alamir et al. (2024) (34)	Cultural, societal, and religious sensitivities were reported as an obstacle to the vaccine by the parents.	Religion and culture are barriers to HPV vaccine uptake
Gulifeiya Abuduxike et al. (2022) (22)	Lack of spouse support is a barrier to vaccinating children.	Lack of recommendations
Alhusayn et al. (2022) (36)	The main barrier is marked by the insufficiency of information regarding HPV, vaccination, and awareness of HPV and its vaccine.	Lack of HPV vaccine information
Tobaiqy et al. (2023) (37)	The most cited excuse for not getting the vaccine was ignorance of the significance of HPV immunization.	Lack of HPV vaccine information
Azh et al. (2021) (38)	The elevated expense of vaccinations, economic challenges, and insufficient awareness were serious obstacles to HPV immunization.	Lack of HPV vaccine information Cost barriers
Ortashi et al. (2014) (27), Saqer et al. (2017) (19), Husain et al. (2019) (39), Jradi and Bawazir (2019) (14)	The significant apprehension that parents seem to have with vaccination of their children could be due to the HPV vaccine’s negative effects and safety.	Lack of safety of the vaccine
Husain et al. (2019) (39)	83.6% of the participating parents in their research were worried about the safety of HPV vaccines and required reassurance that the immunization would provide immunity to an infection with HPV.	Lack of safety of the vaccine
Abou El-Ola et al. (2018) (20)	The same issue, in which 11.7% of the participants in their research believe that the vaccine is not safe, and 2.9% believe it is not practical. However, most of the articles show that most participants are in favor of vaccination	Lack of safety of the vaccine
Saqer et al.’s (2017) (19)	40% of them owed their concern to the lack of knowledge about the vaccine.	Lack of HPV vaccine information
Jradi and Bawazir (2019) (14)	The primary concern for their participants regarding the HPV vaccine was that a few people they knew had it.	Lack of HPV vaccine information
Kilic et al. (2012) (28)	Because they were not properly educated about the HPV vaccination, only 45.5 and 44.9% of mothers and fathers, respectively, were ready for immunization of their daughters against the virus. 23.8 and 25% of mothers and fathers were worried about possible side effects the vaccine might cause.	Lack of HPV vaccine information Lack of safety information about the vaccine
Abuduxike et al. (2022) (22)	One obstacle to kids’ vaccination is a spouse’s lack of support.	Spouse’s lack of support
Shibli and Rishpon (2019) (29)	Less than half of the participant mothers believed the vaccine was safe, and 56.9% believed it had harmful side effects.	Lack of safety of the vaccine
Alhusayn et al. (2022) (36)	The primary obstacle is the dearth of information about HPV infection, vaccination, and awareness of HPV and its vaccine	Lack of HPV vaccine information
Alnaeem et al. (2023) (23)	The primary obstacles to immunization were worries about side effects and vaccine efficacy.	Lack of safety of the vaccine
Bin Alamir et al. (2024) (34)	Parents cited cultural, sociological, and religious sensitivity as an obstacle to the HPV vaccine.	Religion and culture are obstacles to vaccine acceptance
Tobaiqy et al. (2023) (37)	Ignorance of the significance of HPV vaccination was cited as the main objection to the vaccine.	Lack of HPV vaccine information
Azh et al. (2021) (38)	Barriers include the high cost, financial difficulties, and ignorance.	Decreased information regarding the HPV vaccine. Cost barriers

#### Limited vaccine information and recommendations

Most studies showed that a lack of vaccine information is the main perceived barrier to HPV vaccine uptake. Factors such as withholding information (14), fear of sexual promiscuity in children (39), insufficient understanding of the importance and harm of vaccination (26, 37, 42), and lack of knowledge and awareness of the necessity of vaccination are also perceived barriers (14, 19, 28, 29, 30, 36, 45). Lack of awareness regarding the personal risk of HPV infection also constitutes a barrier (30). Less knowledge of vaccine safety and efficacy was a potential barrier to HPV vaccination (14, 19, 20, 23, 25, 27, 39, 44).

#### Associated costs

Studies also found that the cost of the HPV vaccine was significantly associated with uptake. Based on the findings, most parents lamented that the cost of the vaccine made it impossible to acquire, constraining its affordability (12, 25, 38, 39, 41).

#### Religion and culture

Accordingly, religion and culture have been presented as significant barriers to HPV vaccine uptake and tolerability (19, 27, 34, 40). Most parents, who were the main participants, attributed their cultural beliefs and social and religious sensitivities as reasons why they did not consider taking the HPV vaccination for their children.

#### Lack of support

Studies indicated that HPV vaccination uptake is significantly influenced by support, such as from the Ministry of Health (MOH), and belief in vaccine efficacy (23). Therefore, a lack of support was perceived as a barrier to HPV vaccination in children (12, 18, 22). Other barriers to HPV vaccine uptake included limited vaccine availability and access (16), lack of time (14, 17, 28, 35, 36, 38), and a blurred understanding of vaccine significance (18), possibly due to inadequate recommendations from health practitioners or spouses.

### Perceived benefits of HPV vaccination

Perceived benefits were the returns people achieved by changing or adopting a particular health behavior. The study found that the main perceived benefits of HPV vaccination were disease prevention and knowledge of the HPV vaccine. Table 9 showed the main perceived HPV vaccination benefits themes.

**Table 9:** Main perceived HPV vaccination benefits.

Study	HBM concept: Perceived benefits	Themes
	*All authors accept that the HPV vaccine is essential for preventing cervical cancer.*	*Prevention of disease*
Alnafisah et al.’s (2019) (18)	82.2% of women find HPV vaccination essential, and 68.6% believe it helps prevent cervical cancer.	Prevention of disease
Anfinan (2019) (25)	81.4% of their research participants knew that Pap smear tests could help determine the presence of an HPV infection, so 41.2% of them agreed to take the vaccine.	Prevention of disease
Anfinan (2019) (25)	Some women thought of the HPV vaccine as a treatment for HPV infection and not a precaution, which was one of the perceived benefits of the vaccine.	Misconception: vaccine perceived as treatment for existing HPV infection rather than prevention—not a perceived benefit.
Ortashi et al. (2014) (27)	They reported that 68.6% of the women in their research were aware of the ability of the HPV vaccination to prevent cervical cancer.	Prevention of disease
Alzahrani et al. (2017) (12)	Discussed its efficiency, benefits, and safety.	
Alrajeh et al. (2020) (26)	24.2% of their participants agreed that an HPV vaccination could protect against cervical cancer.	Prevention of disease
Shibli and Rishpon (2019) (29)	47.6% of the parents in the research believed that their children might get HPV at some time in their lives, so 62% believed it to be effective, while 47.6% thought it was a safe option.	Prevention of disease
Kilic et al. (2012) (28)	72.3% of the mothers and 62% of the fathers showed their acceptance of vaccinating their child because they believed it would protect against cervical and HPV infection.	Prevention of disease
Levent et al. (2021) (41)	76% of the parents who considered getting their children vaccinated had daughters, believing that it would protect them from cervical cancer.	Prevention of disease
Alyahya et al. (2024) (45)	34.2% of parents believed that the HPV vaccine prevents cervical cancer, and 39.9% of parents believed that the vaccine prevented genital warts.	Prevention of disease and warts
Tobaiqy et al. (2023) (37)	Only a small proportion of participants had knowledge about the benefits of HPV vaccination to prevent genital warts and CC.	Limited knowledge of vaccine benefits—knowledge finding, not direct perceived benefit.
Ghojazadeh et al. (2012) (24)	Mothers’ knowledge of the advantages of HPV vaccination scored better on average than fathers’.	Mothers had greater knowledge of vaccine benefits than fathers—knowledge finding; education is a modifying variable.
Alshehri et al. (2023) (15)	44.5% of parents know about the benefits of HPV vaccination and therefore have the intention to vaccinate their children.	Knowledge of vaccine benefits associated with intention to vaccinate—knowledge finding
Çelik et al. (2021) (33)	Knowledge about vaccine benefits was strongly related to acceptance of the HPV vaccine.	Knowledge of vaccine benefits strongly associated with vaccine acceptance—knowledge finding, not direct perceived benefit measurement.

Entries labeled ‘knowledge finding’ reflect parental knowledge or awareness of HPV vaccine benefits rather than the subjective belief that vaccination will provide personally relevant benefits. The Anfinan (25) entry reflects a misconception about vaccine function and is classified accordingly rather than as a perceived benefit.

#### Disease prevention

The authors agreed on the importance of the HPV vaccine for preventing CC and genital warts (12, 18, 27, 29, 37, 41, 45). They believe that taking tests helps to determine the source of HPV infection (25). One finding warrants specific attention: the belief held by some women that the HPV vaccine treats an existing HPV infection (25) is more accurately classified as a misconception about vaccine function rather than a perceived benefit (25), and is reported as such in Table 9. Besides CC protection, it was also found that HPV vaccination prevented genital warts (37, 45).

#### Educational attainment and HPV vaccine knowledge

Studies reported that educational attainment and understanding of the HPV vaccine were associated with perceived benefits of HPV vaccination (24). It should be noted that findings describing parents’ knowledge of vaccine benefits—rather than their subjective belief that the vaccine will provide personally relevant benefits—are better classified as knowledge findings and are labeled accordingly in Table 9. Parents who had knowledge of vaccine benefits were more likely to vaccinate their children or hold positive attitudes toward HPV vaccine uptake (15, 33).

### Cues to action as HPV vaccination measure

Of the 28 studies that reported on cues to action in relation to HPV vaccine uptake, vaccine-related doctors’ advice and vaccine-related MOH guidelines coupled with vaccine education were presented as the main themes influencing cues to action (Table 10).

**Table 10:** Main themes influencing cues to action.

Study	HBM concept: cues to action	Themes
Çelik et al. (2021) (33)	Prescription of the HPV vaccine by a professional medical doctor was considered to have a positive impact on the uptake of vaccination	Cue to action for uptake of HPV vaccine-related doctor advice.
Alnaeem et al. (2023) (23)	62.9% of parents expressed worry that their physicians did not discuss the vaccine, and that if they did, they would be inclined to vaccinate their children.	Cue to action for uptake of HPV vaccine-related doctor advice.
Ghojazadeh et al. (2012) (24)	Involvement of healthcare professionals and the MOH can improve HPV vaccination acceptance among parents. Therefore, healthcare professionals should inform parents about HPV vaccination in healthcare settings.	Cue to action for uptake of HPV vaccine-related doctor advice.
Abuduxike et al. (2022) (22)	Policies and guidelines from relevant ministries can improve HPV vaccination uptake.	Cue to action for uptake of HPV vaccine-related guidelines from MOH.
Alshehri et al. (2023) (15)	If the MOH sent proper notice, parents would vaccinate their children. Awareness campaigns from relevant ministries can also enhance parents’ acceptance of vaccinating their children.	Cue to action for uptake of HPV vaccine-related guidelines from MOH.
Abou El-Ola et al. (2018) (20)	To increase HPV vaccination rates, public education campaigns and a combined strategy to address the identified challenges are recommended.	Educational intervention recommended, primarily knowledge-building rather than a direct cue to action
Alherz et al. (2024) (21)	To increase the acceptability of HPV vaccination, parents’ knowledge must be increased. The Saudi Ministry of Health’s proposed initiatives would also increase public awareness and improve the HPV vaccine’s acceptance rate.	Cue to action for uptake of HPV vaccine-related guidelines from MOH.
Elissa et al. (2024) (31)	To raise parental acceptability of the vaccine among their child, educational interventions are required to raise parental knowledge, awareness, and attitudes around HPV.	Educational intervention recommended—primarily knowledge-building rather than a direct cue to action.
Alnaeem et al. (2023) (23)	To increase vaccination uptake, a focused awareness campaign and better parent-provider communication are required.	Cue to action for uptake of HPV vaccine-related doctor advice.
Bin Alamir et al. (2024) (34)	Specifically designed training programs and medical specialists are essential to increase HPV vaccination rates and address knowledge gaps. To address the various problems surrounding HPV vaccination and raise community vaccine knowledge, the research recommended a variety of multimodal approaches, such as policy strategies, focused awareness campaigns, and educational interventions.	Cue to action for uptake of HPV vaccine-related guidelines from MOH.
Seven et al. (2015) (17)	Educate both men and women about HPV and vaccination, and support efforts to raise awareness and improve HCP knowledge to boost uptake.	Educational intervention recommended—primarily knowledge-building rather than a direct cue to action.
Alaamri et al. (2023) (35)	Social media campaigns and educational initiatives are approaches that should be implemented. Clinicians must be engaged, as their recommendations influence parents’ decisions to vaccinate their children.	Cue to action to uptake HPV vaccine-related guidelines from MOH.
Alkalash et al. (2022) (30)	The advantages of screening programs and the need to educate women concerning the risk factors associated with cervical cancer. To motivate individuals to be vaccinated against HPV, practitioners should educate them about the benefits of HPV vaccination. Public awareness and knowledge in Saudi Arabia would rise with a well-thought-out educational campaign on the advantages of screening and the vaccine.	Educational intervention recommended—primarily knowledge-building rather than a direct cue to action.
Alshehri et al. (2023) (15)	Culturally sensitive health promotion campaigns for the HPV vaccination should highlight the vaccine’s advantages and disadvantages for women.	Cue to action to uptake HPV vaccine-related guidelines from MOH by cultural response.
Alhusayn et al. (2022) (36)	To boost Saudi parents’ acceptability of HPV vaccination, there is a need for a practical awareness course and improved HPV-related education.	Cue to action to uptake HPV vaccine-related guidelines from MOH by increasing education about the vaccine.
Tobaiqy et al. (2023) (37)	To minimize the risks of cervical cancer, programs that encourage parental knowledge, acceptability, and willingness to receive HPV vaccinations for particular demographics must be developed.	Cue to action to uptake HPV vaccine-related guidelines from MOH
Azh et al. (2021) (38)	Policymakers should encourage this approach, and health professionals need to remind teenagers and their parents about the positive effects of the HPV vaccine.	Cue to action to uptake HPV vaccine-related guidelines from MOH and doctors.
Alnafisah et al. (2019) (18)	In order to eradicate unfavorable attitudes and beliefs around HPV vaccination, awareness programs are required to increase understanding and boost participation.	Educational intervention recommended—awareness programs primarily targeting knowledge and attitudes rather than functioning as a direct cue to action.
Anfinan (2019) (25)	To encourage doctors to take an active role in the battle against cervical cancer and HPV infection, more targeted educational interventions are essential. By demonstrating the vaccine’s safety, accessibility, and efficacy, and by providing clear information on the immunization process and objectives, such initiatives should help demystify HPV and support an HPV prevention strategy.	Cue to action to uptake HPV vaccine-related guidelines from MOH
Husain et al. (2019) (39)	Education on HPV infection and the function associated with the HPV vaccine is necessary for Bahraini society. Future awareness efforts may focus on filling some of the important knowledge gaps around HPV that the study identified. To increase vaccination acceptability, it is necessary to prioritize addressing men’s lack of knowledge, which may present particular difficulties.	Cue to action to uptake HPV vaccine-related guidelines from MOH
Jassim et al. (2018) (40)	In order to avoid cervical cancer, there needs to be a long-term effort to raise knowledge. It is also important for basic healthcare centers across the country to implement a community-based screening program.	Cue to action to uptake HPV vaccine-related guidelines from MOH
Alrajeh and Alshammari (2020) (26)	Given recent reforms in the Saudi health system that prioritize prevention, health policymakers need to establish a nationwide prevention plan to boost the adoption of the HPV vaccination and cervical screening strategy. Using a variety of platforms and relevant interactive media, behavioral and informational interventions may enhance vaccination comprehension and knowledge.	Cue to action to uptake HPV vaccine-related guidelines from MOH
Ortashi et al. (2014) (27)	Parents’ knowledge and attitudes can be improved through mass media campaigns and healthcare-derived interventions, which should be developed in the UAE.	Cue to action to uptake HPV vaccine-related guidelines from MOH
Saqer et al. (2017) (19)	Discuss how government and MOH recommendations were helpful and convincing for parents participating in their research regarding the vaccination of their daughters.	Cue to action to uptake HPV vaccine-related guidelines from MOH
Kilic et al. (2012) (28)	Providing accurate and appropriate information about vaccination is essential not only for adolescent girls, the target population, but also for their parents to increase vaccine uptake.	Cue to action to uptake HPV vaccine-related guidelines from MOH
Shibli and Rishpon (2019) (29)	To enhance beliefs about HPV vaccination and eventually raise its acceptance rate, a special program that clarifies misunderstandings and highlights its benefits and safety should be developed. To enhance Arab parents’ general awareness and comprehension of the HPV vaccine, the priority of these programs should be tailored to the area in which they are carried out. Additionally, emphasis should be placed on improving the views of Jewish parents regarding the HPV vaccine.	Cue to action to uptake HPV vaccine-related guidelines from MOH
Levent et al. (2021) (41)	To increase immunization rates, all parents and adolescents enrolling at any health center should be informed about the vaccine. Just informing the general public of the need to raise vaccination rates is not sufficient.	Cue to action to uptake HPV vaccine-related guidelines from MOH
Levent et al. (2021) (41)	Governmental assistance should be included in the vaccine schedule, and healthcare professionals’ expertise in this area should be consistently improved.	Cue to action to uptake HPV vaccine-related guidelines from MOH
Alsaad et al. (2012) (42)	Before implementing any HPV vaccination program, more must be done to educate Syrians. Mothers, the general public, and healthcare professionals must all be educated as the main focus of public health strategies.	Cue to action to follow MOH HPV vaccine guidelines by increasing education for mothers.
Abou El-Ola et al. (2018) (20)	To increase HPV vaccination rates, awareness campaigns and an integrated strategy that addresses the identified obstacles would be advisable.	Cue to action to uptake HPV vaccine-related guidelines from MOH
Hendaus et al. (2021) (43)	Strategies and interventions must focus on raising parents’ knowledge and educating them, as parents’ beliefs are seen as essential objectives for societal health interventions.	
Al Alawi et al. (2023) (44)	Oman requires efforts to introduce HPV education at both the school and public levels, and it also requires a national strategy for the HPV vaccine.	Cue to action to uptake HPV vaccine-related guidelines from MOE in schools by the national HPV vaccination program.
Alyahya et al. (2024) (45)	Public pharmacies may help increase coverage and perception of HPV vaccination, and targeting the parents of teenagers is essential for immunization campaigns.	Cue to action to uptake HPV vaccine-related education for parents and adolescents.

Entries labeled ‘Educational intervention recommended’ reflect study recommendations for knowledge-building campaigns or awareness programs rather than direct external prompts to initiate vaccination. These recommendations primarily operate on perceived susceptibility, severity, and benefits rather than functioning as cues to action in the strict HBM sense. Entries labeled ‘Cue to action’ reflect direct external prompts—such as physician recommendations, MOH notifications, school-based vaccination delivery, or formal government communications—that prompt vaccine initiation regardless of prior knowledge level.

#### Physician advice

Cues to action were the HBM construct most consistently and robustly supported across included studies, with physician recommendation emerging as the single most powerful external prompt for parental vaccine uptake. The finding from Alnaeem et al. (23) that 62.9% of parents stated they would vaccinate their children if their physician recommended it (23) encapsulates a pattern repeated across multiple countries and settings: where healthcare provider communication was absent, parents frequently cited this as their primary reason for non-vaccination; where it was present, it was strongly associated with acceptance. This suggests that in ME contexts, physician recommendation functions not merely as information transfer but as a legitimizing cue that resolves parental uncertainty about safety, necessity, and cultural acceptability.

Ministry of Health guidelines and national-level communications represented the second major cue-to-action theme. Studies from Saudi Arabia and the UAE, the only ME countries with established national HPV vaccination programs, consistently reported that MOH endorsement and school-based delivery increased perceived vaccine legitimacy and reduced parental hesitancy (21, 35). This policy-level cue was frequently cited alongside physician recommendation, suggesting that the two work synergistically: national program endorsement establishes population-level credibility, while individual provider recommendation converts that credibility into parental action.

However, a distinction must be drawn between cues to action that directly prompt vaccination, such as a physician’s explicit recommendation, a school vaccination notice, or an MOH notification, and educational campaigns that primarily increase knowledge. While the latter are valuable and were widely recommended across included studies, they operate primarily on perceived susceptibility, severity, and benefits rather than functioning as cues to action in the strict HBM sense. Culturally responsive campaign design was specifically highlighted as essential in ME contexts (15), where campaigns framed around religious permissibility and family protection rather than individual risk may be more effective in prompting parental action.

### Self-efficacy as an HPV vaccination measure

Self-efficacy—defined in the HBM as a parent’s confidence in their ability to arrange, obtain, and complete HPV vaccination for their child—was the construct least directly evidenced across the 33 included studies. After carefully re-examining each study’s original measurement approach, direct evidence was limited to two studies. Shibli and Rishpon (29) found that self-efficacy was positively associated with maternal acceptance of HPV vaccination in Israel (29), and Bin Alamir et al. (34) reported that educated parents demonstrated greater confidence in vaccine decision-making in Saudi Arabia (34). These findings are consistent with the HBM prediction that self-efficacy moderates the relationship between motivation and behavior, but they represent a narrow evidentiary base.

Several findings initially considered under this construct were more accurately reclassified upon closer examination. Parents who were healthcare professionals and demonstrated greater vaccine confidence are better characterized as a modifying variable effect of occupational knowledge (15, 32). The association between physician advice and parental vaccine confidence is more appropriately classified as a cue to action (23). The belief that children were too young for vaccination represents a knowledge misconception or perceived barrier rather than low self-efficacy (20). Additionally, parental unwillingness to vaccinate is an attitudinal outcome, not a measure of confidence in completing vaccination (28).

The overall scarcity of direct self-efficacy evidence is itself a meaningful finding. In contexts where cost, vaccine availability, male guardianship, and provider access all impose structural constraints on parents’ ability to act on vaccination intentions, self-efficacy is likely to be a more important determinant of behavior than the current literature captures. Future research using validated self-efficacy measures in ME populations is therefore a priority gap identified by this review (Table 11).

**Table 11:** Self-efficacy themes.

Study	HBM concept: self-efficacy	Themes
Akca et al. (2022) (32)	Reported that mothers who were healthcare professionals had self-efficacy regarding vaccinating their daughters.	Mothers who were healthcare professionals had greater vaccine confidence—a modifying effect of occupation, not a direct self-efficacy measure.
Alshehri et al. (2023) (15)	Healthcare professionals had the self-efficacy to vaccinate their children.	Healthcare professional parents had greater vaccine confidence—a modifying variable, reclassified from self-efficacy.
Tobaiqy et al. (2023) (37)	The main reason behind low efficacy was insufficient awareness of the significance of HPV vaccination. Awareness of the significance of HPV vaccination seems to help improve self-efficacy.	Insufficient awareness of HPV vaccination significance—a knowledge gap; more accurately a barrier or knowledge finding than self-efficacy.
Alkalash et al. (2022) (30)	Participants who received information from physicians about HPV vaccination had high awareness scores and self-efficacy.	High self-efficacy is associated with advice from healthcare professionals.
Bin Alamir et al. (2024) (34)	Educated women exhibited higher levels of awareness and self-efficacy with HPV vaccination compared to men.	Higher efficacy is associated with high information.
Shibli and Rishpon (2019) (29)	Attitude and self-efficacy were positively related to the mother’s acceptance of the vaccine.	Self-efficacy is associated with acceptance of the HPV vaccine.
Saqer et al. (2017) (19)	Parents’ self-efficacy was associated with authorities’ recommendations for vaccination.	Parental vaccination readiness was associated with authoritative recommendations—a cue to action; reclassified from self-efficacy.
Jassim et al.’s (2018) (40)	37.7% of research participants believed their kids were not old enough to get vaccinated and were confused that it was only suitable for a specific age.	Belief that children are too young for vaccination—perceived barrier or knowledge misconception; reclassified from self-efficacy.
Kilic et al. (2012) (28)	Of 301 respondents, 4.9% of fathers and 4.3% of mothers did not consider the HPV vaccine beneficial, which is why they were unwilling to take it.	Parental unwillingness to vaccinate—an attitudinal outcome; not a self-efficacy measure; reclassified

Only Shibli and Rishpon (29) and Bin Alamir et al. (34) provide findings that most closely reflect parental self-efficacy as defined by the HBM. All other entries are mapped interpretively; several are more accurately classified as cues to action, perceived barriers, modifying variables, or attitudinal outcomes, and are labeled accordingly. Direct evidence of parental self-efficacy as a distinct construct was limited across the included studies.

### Other factors

#### Modifying variables

Across the studies included, sociodemographic characteristics, such as age, sex/gender, educational level, income, occupation, and nationality, were established as modifiable factors that affect the relationship between HBM beliefs and vaccination behaviors. Women who had a family history of cervical cancer had a more positive attitude toward the vaccine (28). A significant correlation was found between a male spouse’s educational attainment and their influence on their wife’s or daughter’s vaccination decision (19), though a negative correlation between parental knowledge and agreement to vaccinate daughters was also noted (18).

#### Sociocultural and contextual factors beyond the HBM

Male guardianship as a structural/sociocultural factor was present across various studies (12, 14, 18, 19, 40). Age and education are typical HBM modifying variables, while the role of a male guardian needs to be viewed as a structural or environmental condition that impacts the external decision-making context in which mothers develop and execute their decisions to vaccinate. Other contextual factors, such as cultural norms, religious sensitivities, and stigma associated with HPV transmission through sex, persisted outside the usual tenets of the individual-level HBM.

### Trust and attitudinal factors

Some participants found MOH statements untrustworthy (29). In Jordan, 19.2% of parents believed the HPV vaccine’s efficacy was unclear (45). While HPV vaccine uptake on its own did not significantly influence or change vaccination rates in individuals, it did show that HPV vaccine uptake in individuals increased with increases in trust in the MOH, indicating that HPV vaccine uptake needs to be reinforced by regular communication and accessible delivery via the MOH (29).

### Knowledge and information sources

Of 1,403 parents, 22.6% had heard of HPV infection and vaccination, with digital platforms being the most prevalent information source (44). In Lebanon, parents were more likely to receive HPV vaccine information from doctors than from parent education television sessions (16). Several factors were identified as potential risk factors for HPV infection, such as smoking and early marriage (45). The study revealed that 13.5% agreed that vaccinations should be given to girls when they are 9 years old (26) and 45% agreed that HPV should be administered prior to their children having knowledge of sexual relations (43), with more than 90% agreeing that HPV information should be made available to adolescents (39).

#### The attitude of parents toward HPV vaccination uptake

Most parents in the included studies demonstrated positive attitudes toward HPV vaccination. However, a consistent distinction must be drawn between willingness and actual uptake, as most included studies measured only the former. Regarding willingness to vaccinate (W): between 58 and 94.6% of participants across studies indicated awareness and general support for vaccination; Alnafisah et al. (18) reported that 70.8% of mothers consented to vaccinate when their daughters reached school age (W); Anfinan (25) reported that 77.6% of parents consented to vaccination (W); Jassim et al. (40) reported 90.9% willingness (W); and Husain et al. (39) reported 76% willingness (W). In contrast, actual uptake rates were substantially lower where reported: Ortashi et al. (27) (UAE) reported that only 9.8% of mothers had actually vaccinated their daughters (U); and Çelik et al. (33) (Turkey) reported that HPV vaccinations had been given to children by only two parents in a sample of 1,000 (U). In Turkey, willingness among mothers was 45.5% and among fathers 44.9% (W) (28), while in Jordan only 35% of parents trusted the vaccine enough to consider it (W) (45). In Lebanon, 60% of the 185 parents who were aware of the vaccine had no plans to use it (W) (16). These findings collectively suggest a substantial willingness–uptake gap across the region, with attitudinal readiness consistently exceeding actual vaccination behavior.

Among the included studies, most assessed parents’ willingness to vaccinate, while only a minority reported actual HPV vaccination uptake, typically defined as parent-reported receipt of at least one dose. Willingness to vaccinate was generally higher than documented uptake where both were reported, indicating a potential willingness–behavior gap with implications for designing interventions that target both beliefs and structural barriers. Table 5 clarifies which outcome each study measured: the outcomes of willingness to vaccinate and actual vaccination uptake.

## Discussion

This systematic review is the first to synthesize evidence specifically from studies conducted within ME countries among parents residing in the region, examining parental knowledge, beliefs, and attitudes toward HPV vaccination through the lens of the HBM. Unlike the review by Netfa et al. (8), which focused on Arabic-speaking immigrant parents in Australia, the present review captures findings from 17 ME countries and reflects the health system, policy, and sociocultural contexts of parents living within the region. The review confirms and substantially extends earlier regional work by demonstrating that low HPV knowledge, safety concerns, cost barriers, and cultural and religious influences are pervasive across the ME, while also identifying important country-level variation that has not been previously characterized. In addition to that, the review explain how does the HBM explain or predict HPV vaccination acceptance in ME countries, and how do government policies in ME countries support HPV vaccine acceptance. Structuring the discussion around these research questions would make the key takeaways clearer.

Parental acceptability of HPV vaccination in Middle Eastern nations was associated with a complex interaction of insufficient knowledge, different beliefs, social issues, and practical obstacles. In the analyzed research, a majority of parents indicated a lack of knowledge about HPV, its association with CC and the vaccine’s purpose, with significant proportions either unwilling or confused regarding vaccinating their children. It is important to note that most included studies measured willingness or intention to vaccinate rather than actual uptake; where both were reported, actual uptake was consistently lower than expressed willingness, highlighting a willingness–uptake gap that warrants targeted behavioral interventions beyond attitude change alone. Safety concerns, doubts about effectiveness, cost, perceived lack of personal risk, and limited physician recommendations were the most frequently reported barriers, while trust in health authorities, higher education, and clear information from healthcare professionals tended to support more favorable attitudes and greater uptake of vaccination. Cultural and religious considerations also contributed to controversy: in several studies, parents perceived HPV vaccination as morally unnecessary, worried that it might implicitly condone premarital sexual activity, or expressed uncertainty about religious permissibility and the association with a sexually transmitted infection. For instance, Netfa et al.’s 2020 review found that economic concerns, safety concerns, cultural and religious beliefs, negative attitudes, and lack of knowledge of HPV and its vaccine were the most common factors influencing the acceptability of HPV vaccination among Arabic-speaking mothers in Australia (8). Male guardianship represented a structural factor reported across multiple studies (12, 14, 18, 19, 40), in which mothers’ vaccination decisions were contingent on or constrained by male partner approval. This is analytically distinct from individual-level HBM barriers; it operates at the level of household decision-making authority and requires sociocultural and structural analytical frameworks beyond the HBM to adequately interpret. Vaccination costs and advice from a doctor or family also influenced parents’ decisions (47). A WHO Eastern Mediterranean Regional Office systematic review found that despite low to moderate knowledge about HPV infection, some countries in the ME have moderate to high levels of HPV vaccine acceptability (48). However, cultural and religious sensitivity has led to decreased inclusion of HPV in national immunization programs due to fear of public backlash (49).

The HBM provides a practical organizing framework for interpreting the reported associations between parental beliefs and vaccination intentions and behavior in ME settings. Because 32 of 33 included studies used cross-sectional designs, the HBM should be understood here as a theoretical lens for interpretation rather than a tool with established predictive value. Perceived susceptibility to HPV and the perceived severity of its effects were frequently minimized: parents often thought their children were at low risk for HPV because they believed they would wait until marriage to have sex or due to strong religious beliefs. This made them less likely to feel they needed vaccination. In line with HBM predictions, parents’ perceived benefits and uptake of vaccination were consistently higher when they had a thorough understanding of how the HPV vaccine helps prevent genital warts and cervical cancer. Perceived barriers, including fear of side effects, safety, financial cost, access difficulties, and stigma, were strongly and repeatedly associated with hesitancy or refusal, again reflecting core HBM constructs. Cues to action, such as clear recommendations from doctors, vaccination offers at schools, and visible support from Ministries of Health, were all reported as essential factors that increased people’s likelihood of accepting the vaccine. Self-efficacy, as evidenced by parents’ confidence in securing and completing their children’s vaccine series, was affected by cost, availability, and the clarity of information, and was associated with uptake when these factors were considered. Critically, several factors identified in this review are not adequately captured by traditional HBM domains. Male guardianship authority, pervasive restrictions on discussions of sexual health, and general vaccine mistrust operate at sociocultural and structural levels that the individual-level HBM was not designed to address. The HBM, therefore, provides a useful but incomplete theoretical framework for this context and should be applied alongside sociocultural and structural analytical models in future research on HPV vaccination in the ME region. This means that the HBM explains many of the differences in acceptance, but it should be used alongside sociocultural and structural frameworks in ME contexts. The stigma associated with STIs and the potential perception of vaccination as unsafe sexual conduct might hinder vaccine uptake (50). Studies on women’s opinions on HPV screening have also shown that religious beliefs can deter vaccine and cervical screening (51). Male parents played a crucial role in children’s vaccination decisions, so targeting them equally in awareness campaigns is essential. Female participants highlighted male guardianship and potential barriers to their daughters’ vaccination, and higher perceived barriers and risks about the harm of vaccines had reduced the scope of vaccination, particularly for HPV vaccines (52). Insurance coverage was another barrier, especially in the United States (52).

Government policies and health system arrangements in ME countries critically shape the environment in which parents form their beliefs and make HPV vaccination decisions. Only a few ME countries, like Saudi Arabia and the United Arab Emirates, have included HPV vaccination in their national programs. These programs usually target teenage girls but sometimes include boys as well. Thus, providing clear official cues to action helps ensure the vaccine is accepted as part of routine child care. In these settings, public funding, organized delivery (e.g., through schools or primary care), and explicit recommendations from Ministries of Health are associated with greater perceived legitimacy and importance of the vaccine and higher reported uptake among parents. On the other hand, where HPV vaccination is still optional, self-funded, or offered mainly by private providers, parents often state that the price, limitations in availability, and an absence of clear government support are reasons why they do not receive it, which supports the perceived barrier found in the HBM. Nevertheless, the pattern across studies suggests that policies which ensure financial coverage, embed HPV vaccination into routine national immunization schedules, involve schools and primary care providers, and explicitly address religious and safety concerns through trusted authorities are likely to strengthen parental acceptance and uptake of HPV vaccination across the region. In contrast, studies showed that 95% of fathers passionately believe in vaccinating their daughters before marriage (12). The majority of male partners were increasingly supporting their wives for Pap smear and HPV vaccination. Public uptake of vaccination increases when recommended by their MOH (19, 27, 46). Similarly, trust in governmental programs played a crucial role in the decision to use the HPV vaccine, as knowledge is influenced by campaigns and government-sponsored programs (53).

Results of the present review support the general trend of international systematic reviews of HPV vaccine uptake. Radisic et al. (54) similarly found that knowledge, family dynamics, and cultural values shape parental decisions, while Wijayanti et al. (55) identified low risk perception and safety concerns as dominant barriers in Southeast Asian contexts, patterns mirrored across ME countries. The specific intersection of male guardianship authority over household health decisions, an extensive religious discourse around sexual health, and the limited presence of government-supported HPV immunization and vaccination programs characterize the ME context. Therefore, successful interventions in this region could include gender-specific, religion-specific, and policy-level components (education, healthcare provider engagement, policy support).

### Strengths and limitations

This is the first paper to synthesize qualitative evidence from many parents across several countries in the ME region. The findings may, to some extent, represent parents’ attitudes and beliefs about HPV vaccine uptake for their children, as people in this region share many commonalities, such as cultural and religious beliefs.

However, this review has certain limitations. As the studies included were conducted in ME countries, the region’s cultural, social, and economic specificities may limit the generalizability of the findings to other populations.

In addition, language constraints were imposed to include only English-language publications, as there would be limited access to professional translation services for others. This is a well-documented type of literature-based systematic review selection bias. Some studies in Arabic, Turkish, Persian, and Hebrew (the main languages used for scientific publication in ME countries) might not have been summarized. This may result in an undue underrepresentation of HPV-related parental research from Iran, Turkey, and non-English-publishing ME countries where HPV-related parental research does exist but is mainly published in national-language journals. Going forward, multilingual search approaches and tools for translation should be emphasized in future reviews to facilitate fuller regional evidence synthesis. These limitations highlight the need for further research that incorporates diverse geographic regions and linguistic inclusivity to provide a more comprehensive perspective. The overall confidence in the review’s conclusions is tempered by methodological limitations in the included studies. Although all studies reported clearly focused aims and acceptable outcome measures, several did not adequately describe how potential confounders were handled, and many lacked sample size justification, which may reduce the internal validity and precision of estimated associations between parental beliefs and vaccination outcomes. In addition, most studies used cross-sectional self-reported questionnaires and convenience samples from single settings, and generalizability was judged to be questionable in around one-third of studies, meaning that the direction of associations (e.g., between knowledge, perceived barriers, and acceptance) is more certain than the exact magnitude or transferability across all Middle Eastern contexts. These limitations suggest that the thematic patterns identified, such as the central role of safety concerns, cost, and healthcare provider recommendation, are likely robust, but effect sizes and their applicability to underrepresented populations should be interpreted with caution.

These quality limitations, along with the prevalence of cross-sectional designs, suggest that the findings identify correlates of HPV vaccination uptake rather than establishing causal relationships.

Although most Middle Eastern countries share customs, traditions, and religious beliefs, they can differ in their insurance and healthcare systems. Because most of the included studies are from Saudi Arabia (13 of 33 studies), it can be argued that the evidence base is heavily focused on a single national context with specific health, legal, and cultural characteristics. This emphasis indicates that the consolidated results, especially regarding obstacles such as cost, awareness, and provider recommendations, are directly relevant to Saudi Arabia and the other Gulf nations, whereas their applicability to other Middle Eastern countries with various insurance frameworks, vaccination policies, minority populations, or economic challenges is limited or different. The review highlights essential regional patterns, but it should be interpreted as providing stronger evidence for the Gulf countries and more preliminary implications for less-represented contexts in other ME countries.

Despite employing a theory-informed narrative synthesis approach that combines inductive coding of study findings with deductive mapping to the HBM regarding cultural and religious influences, almost all included studies are quantitative cross-sectional surveys, with only one qualitative study offering an in-depth exploration of sociocultural meanings. For instance, religion as a barrier and concern about premarital sex are utilized instead of comprehensive narratives, limiting their ability to clarify how norms, gender roles in society, and religious interpretations impact decisions in daily life. The review can identify the presence and approximate prevalence of these concerns; however, it cannot fully explain their nuances or variation across subgroups, and more qualitative and mixed-methods work is needed to deepen understanding.

The majority of the studies included were cross-sectional surveys, and their results were organized into qualitative themes aligned with HBM constructs. Therefore, the review is best interpreted as offering theory-informed insights into the most significant beliefs and contextual factors, rather than providing exact quantitative measures of their effects. This thematic transformation enhances conceptual comprehension while constraining the capacity to quantify the extent of associations or to deduce causality.

### Implications for practice

The Ministries of Health should run awareness campaigns to increase parents’ knowledge regarding HPV and reduce their concerns about the safety and side effects of HPV vaccination through outreach programs and constant engagement with the public.

The next step would be to initiate a vaccination campaign to reduce the prevalence of CC and protect children from cancer.

These initiatives should be complemented by national policy efforts that educate and encourage HPV vaccination as part of a comprehensive approach to reducing HPV-induced cancers and infections. These programs need to assist parents by providing real, evidence-based information that improves their understanding and acceptance of the vaccine. Through education and transparency, these policies can build trust and drive increased HPV vaccination, leading to better public health outcomes.

A notable pattern emerges when the findings are examined by national HPV immunization program status. Saudi Arabia and the UAE, the only two ME countries with established national HPV vaccination programs, showed comparatively higher rates of parental awareness, perceived legitimacy of the vaccine, and MOH-prompted cues to action in included studies. For example, studies from Saudi Arabia frequently reported that MOH recommendations and school-based delivery were cited as strong facilitators of vaccine acceptance (12, 21, 35), and willingness rates in several Saudi studies ranged from 60 to 94.6% (17, 18, 21). In contrast, studies from Lebanon, Turkey, Jordan, and Iran—countries without national programs—more consistently reported cost as a primary barrier, lower familiarity with the vaccine, and the absence of physician recommendations as the main reasons for non-vaccination (15, 16, 28, 45). These country-level differences suggest that embedding HPV vaccination within national immunization schedules, subsidizing costs, and providing structured training for healthcare providers may be the most impactful policy levers for improving uptake across the region. Countries currently without national programs should consider phased introduction supported by community education campaigns tailored to local cultural and religious contexts.

## Conclusion

The awareness of HPV and its potential cancer-causing effects in ME countries was inadequate. Parents were concerned about the lack of information about the virus and vaccines, with barriers including insufficient healthcare providers, safety concerns, vaccine costs, and vaccine availability. There were also gaps in policy and education regarding the virus and vaccination. Cultural, societal, and religious norms influence parents’ perceptions of HPV susceptibility. Parents who were aware of the severity of HPV infection were more likely to accept the vaccine. However, barriers such as high costs, side effects, and a lack of spousal support hinder vaccination. Strategies to improve HPV vaccination uptake included mass medical campaigns, healthcare professional involvement, educational interventions, health insurance coverage, and MOH recommendations. Self-efficacy played a crucial role in enhancing vaccine uptake. This systematic review found that while a substantial proportion of parents across ME countries expressed willingness or intention to vaccinate their children against HPV, actual documented uptake remained considerably lower where it was reported. This willingness–uptake gap underscores the need for interventions that address not only knowledge and attitudes but also structural barriers such as cost, access, and healthcare provider engagement.

## Data Availability

The original contributions presented in the study are included in the article/supplementary material, further inquiries can be directed to the corresponding author.
